# Refining the rheological characteristics of high drug loading ointment via SDS and machine learning

**DOI:** 10.1371/journal.pone.0303199

**Published:** 2024-05-09

**Authors:** Xilong Qian, Kewei Wang, Yulu Ma, Fang Fang, Xiangsong Meng, Liu Zhou, Yanqiong Pan, Yang Zhang, Yehuang Wang, Xiuxiu Wang, Jing Zhao, Bin Jiang, Shengjin Liu

**Affiliations:** 1 State Key Laboratory on Technologies for Chinese Medicine Pharmaceutical Process Control and Intelligent Manufacture, Nanjing University of Chinese Medicine, Nanjing, China; 2 Jiangsu Collaborative Innovation Center of Chinese Medicinal Resources Industrialization, Nanjing, China; 3 College of Pharmacy, Nanjing University of Chinese Medicine, Nanjing, China; 4 Nanjing Hospital of Chinese Medicine Affiliated to Nanjing University of Chinese Medicine, Nanjing, China; 5 Bozhou University, Bozhou, China; 6 Taikang Xianlin Drum Tower Hospital, Nanjing, China; 7 Chemistry and Biomedicine Innovation Center (Chem BIC), School of Chemistry and Chemical Engineering Nanjing University, Nanjing, China; College of Mathematics and Systems Science, Shandong University of Science and Technology, CHINA

## Abstract

This paper presents an optimized preparation process for external ointment using the Definitive Screening Design (DSD) method. The ointment is a Traditional Chinese Medicine (TCM) formula developed by Professor WYH, a renowned TCM practitioner in Jiangsu Province, China, known for its proven clinical efficacy. In this study, a stepwise regression model was employed to analyze the relationship between key process factors (such as mixing speed and time) and rheological parameters. Machine learning techniques, including Monte Carlo simulation, decision tree analysis, and Gaussian process, were used for parameter optimization. Through rigorous experimentation and verification, we have successfully identified the optimal preparation process for WYH ointment. The optimized parameters included drug ratio of 24.5%, mixing time of 8 min, mixing speed of 1175 rpm, petroleum dosage of 79 g, liquid paraffin dosage of 6.7 g. The final ointment formulation was prepared using method B. This research not only contributes to the optimization of the WYH ointment preparation process but also provides valuable insights and practical guidance for designing the preparation processes of other TCM ointments. This advanced DSD method enhances the screening approach for identifying the best preparation process, thereby improving the scientific rigor and quality of TCM ointment preparation processes.

## Introduction

Ointment stands as one of the commonplace topical preparations utilized in clinical practice, serving the treatment of various symptoms like burns, trauma, inflammation, and skin diseases [1–4]. Comprising two components, namely the drug and ointment base, the ratio of these constituents directly impacts the rheological properties of the ointment and subsequently influences its extrusion, extensibility, and stability [5]. Therefore, optimizing the ointment’s preparation process holds significant relevance for ensuring its high quality and improving the overall user experience.

The concept of rheology, first introduced by the American physicist Bingham in 1928, primarily focuses on studying the deformation and flow behavior of matter under external forces. It finds wide application in fields such as chemical engineering, biomedical engineering, and the food industry [6–8]. When it comes to semisolid preparations like ointments, their relevant characteristics can be effectively characterized using a rheometer, which proves integral to the formulation process and the application of ointments. By combining sensory evaluation and rheological parameters, the use experience can be quantitatively described, offering a more comprehensive understanding of sensory attributes and facilitating an intuitive evaluation of the preparation process [9].

To optimize the formulation process, it is imperative to elucidate the relationship between key parameters (such as ingredient ratio and stirring speed) and the objectives of process optimization. However, the choice of an appropriate experimental design method is crucial. Traditional experimental design methods, such as partial factor design and Plackett-Burman design, often only consider two levels of factors (high and low), failing to detect potential nonlinear relationships in the system. Even when these traditional methods incorporate a central point to detect nonlinear relationship, they are unable to identify the factors responsible for the observed effects [10]. In order to obtain a better understanding of the process and optimize it more effectively, a more scientific and comprehensive experimental design method, Definitive Screening Design (DSD), can be employed. DSD considers three levels of factors (high, medium, and low), enabling the identification of nonlinear relationships and important factors without any confounding terms reaching the second order [11]. Ultimately, DSD helps to discover and comprehend the key factors that impact the process, leading to improved efficiency and reliability.

Machine learning serves as a problem-solving technique that leverages the power of artificial intelligence to swiftly uncover hidden rules and trends within data [12]. For the ointment preparation process, there are usually many parameters that need to be adjusted, such as temperature, pressure, reaction time, etc. Traditional methods may involve trial and error and experience, while machine learning technology can automate this process [13,14]. They learn the model by collecting and analyzing a large amount of data, and then optimize it according to the objective function. Machine learning can accelerate the optimization process, reduce test costs, reduce resource waste and improve product quality; It can deal with a large number of data and complex parameter space, and find the correlation that may be ignored [15]. It enables us to find the best parameter settings more quickly, and better understand and use the information in the data. This approach provides more rational solutions to problems and finds widespread applications in domains like medicine, finance, and engineering [16–18].

The formulation of our ointment is derived from the renowned traditional Chinese medicine doctor, Professor Yehuang Wang, from Jiangsu Province, China. The primary ingredients are traditional Chinese medicines, including Corydalis Rhizoma, Radix Sanguisorbae Carbonisata, Natrii Sulfas, Calcine Alunite, Plant soot, and Borneolum, with analgesic, hemostatic, swelling and other effects [19–22]. The aim of this experiment is to establish an experimental framework for optimizing the ointment formulation process, utilizing DSD, decision tree analysis, and Gaussian process optimization. This research framework allows for the accurate and efficient identification of key factors in the ointment preparation process and subsequently constrains their range through decision tree branching. Finally, this constrained range is optimized using Gaussian process optimization. Adopting this method will enhance the optimization of the experimental process, improve its efficiency, and fully unleash the remarkable research and application potential it possesses. In this study, we will present the principles and applications of DSD, decision tree analysis, and Gaussian process optimization, elucidating their roles and value in addressing the challenges we encounter. Through this investigation, we anticipate the provision of an effective integrated approach for the experimental design of ointment and the delivery of more reliable solutions.

## Materials and methods

### Materials

Corydalis Rhizoma obtained from Hubei Jingui Traditional Chinese Medicine Decoction Pieces Co. (A230701, China); Radix Sanguisorbae Carbonisata sourced from Zhangjiagang Green Traditional Chinese Medicine Decoction Pieces Co. (230308, China); Calcined Alunite from Jiangsu Donglian Pharmaceutical Co. (20230301, China); Natrii Sulfas acquired from Zhejiang Zhenyuantang Traditional Chinese Medicine Decoction Piece Co. (20210501, China); Plant soot provided by Bozhou Yonggang Decoction Piece Factory Co. (20220509, China); and Borneolum derived from Jiangsu Zishi Traditional Chinese Medicine Decoction Piece Co. (220603, China). These substances were identified by Professor Liu Xunhong from the Teaching and Research Department of Chinese Medicine Authentication at Nanjing University of Chinese Medicine.

Additionally, petroleum manufactured by Shanghai McLean Biochemical Materials Technology Co. (31HS190112, China); Lanolin provided by Shanghai Yuanye Biotechnology Co. (C15166613, China); Liquid Paraffin obtained from Shanghai Macklin Biochemical Materials Technology Co. (C15315146, China); and Glycerol sourced from Sinopharm Chemical Reagent Co. (20230421, China).

### Preparation of ointment

Weigh the predetermined amounts of ointment base ingredients, namely white petroleum, lanolin, liquid paraffin, and glycerin, into a suitable container. Heat the container in a thermostatic water bath (HH-S4, JMIF, China) to 85°C, allowing the base ingredients to melt and stir evenly. Prepare the drug component of the formulation as a powder and pass it through a 200-mesh sieve (aperture: 75μm ± 4.1μm). Add the drug powder in a proportion determined by the designed method (the menthol should be added below 60°C), and mix the drug and the base thoroughly using a suspended electric stirrer (LC-OES-200SH, LICHEN, China).

### Experimental design and preparation of ointment

The DSD utilizes the conference matrix (*m×m* matrix C) to arrange combinations of experimental factors, satisfying the following equation [23]:

$$C^{\prime }C=(m-1)I_{\left(m\times m\right)}$$ (1)

Here, C represents an *m*×*m* matrix, C’ represents the transpose matrix of C, and I(*m×m*) represents the *m×m* identity matrix.

Depending on the number of factors, there are two scenarios. When the number of factors, m, is even, the experiment is designed using the *m*×*m* conference matrix, with a minimum number of experiments being 2*m*+1. When the number of factors, *m*, is odd, the experiment is designed using the (*m*+1) × (*m*+1) conference matrix, with the last column excluded, resulting in a minimum number of experiments of (2*m*+4) [24]. However, the DSD is a supersaturated design, with second-order orthogonal effects (S1 Fig), and the response follows a normal linear model [25]:

$$yi=\beta_{0}+\sum_{j=1}^{m}\beta_{j}x_{ij}+\sum_{j=1}^{m-1}\sum_{k=j+1}^{m}\beta_{ik}x_{ij}x_{ik}+\sum_{j=1}^{m}\beta_{jj}x_{ij}^{2}+\varepsilon_{i}i=1,\cdots n$$ (2)

The equation is defined as follows: *yi* represents the predicted target, *β*_*0*_ represents the intercept term of the model, *β*_*j*_ represents the influence of the independent variables on the dependent variable, *x*_*ij*_ represents an element in the independent variable matrix indicating the value of *j*^th^ independent variable for *i*^th^ sample, and *ε*_*i*_ denotes random noise in the model. We employed this methodology to establish a correlation between factors and responses (Table 1) and devised 14 distinct ointment preparation processes (Table 2). Obtain the data from three tests of ointment for each scheme, and calculate the average value for further analysis.

**Table 1:** Setting of factors and response limits.

Factor (X)		Response (Y)		Methods (A/B)	
X1	Drug ratio	Y1	Yield stress	A	Medications are added to the molten state of the ointment base.
X2	Mixing time	Y2	High shear viscosity		
X3	Mixing speed	Y3	Low shear viscosity	B	After the base has undergone cooling formation, incorporate the medication.
X4	Dosage of petroleum	Y4	Storage modulus		
X5	Dosage of liquid paraffin				

**Table 2:** Design of deterministic screening experiments.

Sample ID	Drug ratio	Mixing time	Mixing speed	Dosage of petroleum	Dosage of liquid paraffin	Methods
DoE1	35	45	1800	85	10	B
DoE 2	20	5	1800	77.5	10	A
DoE 3	35	25	1050	77.5	7.5	B
DoE 4	20	25	1800	70	5	B
DoE 5	35	25	1050	77.5	7.5	A
DoE 6	50	5	1050	70	10	B
DoE 7	50	25	300	85	10	A
DoE 8	20	5	300	85	7.5	B
DoE 9	50	45	1800	70	7.5	A
DoE 10	20	45	300	70	10	A
DoE 11	20	45	1050	85	5	A
DoE 12	50	5	1800	85	5	B
DoE 13	50	45	300	77.5	5	B
DoE 14	35	5	300	70	5	A

For ease of calculation and preparation, the total weight of each ointment base is 100 g.

### Rheology of the ointment

The rheological behavior of the ointment was evaluated using a rotational rheometer (MCR302, Anton Paar, Austria). The testing was conducted at a temperature of 25°C. A 50mm parallel plate rotor was used for the measurements. First, approximately 2 mL of the ointment sample was placed on the sample stage and excess ointment was trimmed to achieve a gap size of 1.25 mm. The geometric gap between the plates was then adjusted to 1 mm. The sample was allowed to equilibrate at 25°C for 15 minutes before the rheological testing. The rheological data for each sample were measured three times and averaged values were presented along with the standard deviation (SD). The rheological characteristics of the ointment were assessed through several procedures: 1) Oscillatory stress sweep from a strain range spanning from 0.001 to 100 with a frequency set at 1 Hz; 2) Flow curve measuring shear rate from 0.01 to 100 s^-1^, determining low shear viscosity and high shear viscosity, and describing apparent viscosity within the medium to high shear rate range using the power law model: *η = Kγ^n*, where *η* represents viscosity, *γ* represents shear rate, *K* represents consistency coefficient, and *n* represents flow behavior index. When *n*<1, it indicates pseudoplastic behavior; when *n* = 1, it indicates Newtonian behavior; when *n*>1, it indicates dilatant behavior [26]; 3) Temperature sweep, where the sample was equilibrated at 20°C for 2 minutes after loading, and the temperature was then swept from 20°C to 50°C at a rate of 2°C/min with a strain of 0.1% and a frequency of 1 Hz.

### Stepwise regression fitting

Stepwise regression is a multivariate linear modeling method. When using the stepwise regression method, the model will add or remove one independent variable at a time until it reaches the stopping criteria [27]. Each time a variable is added, it will be reevaluated. Variables that have no contribution to the model will be eliminated [28]. The same variable may be added and eliminated multiple times until the "optimal" model is obtained [29]. The foundation of this approach is a multivariate regression equation:

$$yi=\beta_{0}+\beta_{1}x_{1}+\beta_{2}x_{2}+\cdots +\beta_{k}x_{k}+\varepsilon_{i}$$ (3)

In the equation, *yi* represents the predicted value, *x* represents the independent variables, *β*_*0*_ is the intercept, *β*_*1*_, *β*_*2*_, *β*_*3*_… are the slope coefficients, and *ε*_*i*_ is the error term.

Therefore, the importance of variables is determined by iteratively adding or removing independent variables to select the best combination that meets certain criteria. Moreover, the automatic addition or removal of independent variables in the model helps reduce subjective influence, enhancing both the interpretability and predictability of the model.

### Simulation experiment

The multivariate radial stratification method (Table 3) was used to generate data points on different radius layers and simulate multi-dimensional random variables to ensure the diversity and randomness of the data. Firstly, to ensure that the layers selected in each trial are different, the rule of mod(*i*-1, *N* layers) is adopted to determine the number of current runs *i* and the number of layers selected, which is cycled between different layers to achieve diversity. Next, an n-dimensional direction vector is generated on the determined layer. Finally, random distances are determined on demand so that these variables can be scaled and centered.

**Table 3:** Monte Carlo simulation analysis of compartment parameters.

Level	Inner distance	Outer distance
0	0	$\sqrt{d}$
1	$\sqrt{d}$	$\sqrt{d+\sqrt{2d}}$
2	$\sqrt{d+\sqrt{2d}}$	$\sqrt{d+2\sqrt{2d}}$
**…**	**…**	**…**
*i*	$\sqrt{d+(i-1)\sqrt{2d}}$	$\sqrt{d+i\sqrt{2d}}$

### Decision tree modeling

The decision tree is a well-established machine learning algorithm widely applied in diverse domains and data analysis [30,31]. It comprises a hierarchical structure consisting of root nodes, branches, and leaf nodes. By recursively splitting the dataset based on its features into multiple subsets, it serves as a reliable and effective technique for decision-making [31]. In situations where the prior model is flawed, the decision tree can explore variable correlations through fuzzy logic to construct a more suitable model [32,33].

For binary classification problems, the Classification and Regression Trees (CART) algorithm stands out as the most commonly used decision tree algorithm. It classifies the dataset along different paths of the tree structure with each leaf node specific category. The stopping condition of a node that satisfies certain criteria determines the classification result for samples [34,35].

Firstly, we compute prior probabilities (*Prior*_*i*_) for expressing subjective beliefs about parameters:

$${Prior}_{i}=\lambda p_{i}+(1-\lambda )P_{i}$$ (4)

where *p*_*i*_ denotes the prior probability of parent node *i*, *P*_*i*_ represents the posterior probability of parent node *i*, and *λ* is a weighting factor.

Next, using computed Priori values along with sample counts within each node *n*_*i*_, we calculate posterior probabilities (*Prob*_*i*_):

$${Prob}_{i}=\frac{n_{i}+{Prior}_{i}}{\sum \left(n_{i}+{prior}_{i}\right)}$$ (5)

where *n*_*i*_ refers to the number of samples in node *I* and priori indicates prior the probability of response level *i*.

Finally, the Gini index is employed to measure impurity within datasets which enhances adaptability across different nodes while selecting optimal split conditions to optimize the construction of decision trees:

$$Gini(D)=\sum_{i=1}^{m}p_{i}(1-p_{i})=1-\sum_{i=1}^{m}p_{i}^{2}$$ (6)

Where *D* represents the dataset, *Gini(D)* represents the impurity of the dataset, *m* is the number of categories, and *p*_*i*_ is the proportion of samples in category *i*.

### Gaussian process

Gaussian process is a nonparametric Bayesian model used to model the uncertainty of stochastic processes [36]. It describes the probability distribution in function space, and can provide the prediction of function values and the uncertainty of prediction [37]. Gaussian process can be determined by mean function *m*(*x*) and covariance function *k*(*x*, *x’*) [38,39]. The expression is:

f∼GP(m,k) (7)

Where *m* and *k* are used to describe the average function and covariance function of the model. When using the functional estimation model, assume that Y follows a normal distribution with a mean of *μ* (Eq 8) and a covariance matrix (*Σ*) of σ^2^R (σ is random error, Eq 9 defines the R matrix) [38–40].


$$\mu_{i}=m(x_{i})=\frac{1}{4}x_{i}^{2},i=1,\cdots ,n$$ (8)

Where *μ* represents the mean value of the distribution of the generated random vector.


$$r_{ij}=exp(-\sum_{k=1}^{K}\theta_{k}{\left(x_{ik}-x_{jk}\right)}^{2})$$ (9)

Here, *K* represents the number of predicted variables; *θ is the parameter of the kernel function*, *which affects the shape and scale of the kernel function; θ*_*k*_ denotes the *θ* parameter of the *k*^th^ predicted variable; *x*_*ik*_ represents the value of the *k*^th^ predicted variable for object *i*; *x*_*jk*_ represents the value of the *k*^th^ predicted variable for object *j*. Gaussian processes enable rapid and efficient identification of global optima, facilitating further analysis and optimization of the results.

The distribution of the generated random vector (f) can be expressed as a multivariate normal distribution [38,39]:

f∼N(μ,Σ) (10)

Gaussian process can predict the function value of new input by learning the function value distribution and uncertainties in the training data. Once the prediction results of function values are obtained, this information can be used to guide the optimization search process.

### Preliminary stability assessment

1) Place the ointment separately in a temperature-controlled environment at 55°C for 6 hours and at -15°C for 24 hours to test its resistance to heat and cold; 2) Take 10g of the ointment and place it in a centrifuge tube, centrifuge at 2500 rpm for 30 minutes; 3) Allow the ointment to sit at room temperature for two months.

### Micro analysis

During the process of preparing sample slides, it is necessary to evenly apply an appropriate amount of ointment sample onto a glass slide, ensuring that its surface area is similar to that of the cover slip. Subsequently, three prepared slides are placed on the stage of an optical microscope (ZEISS Axio Scope A1 Pol, Germany) for the observation of the ointment sample. In this process, the insoluble solid powder should be clearly visible. Once the images are obtained, particle measurements are conducted using ImageJ (NIH, US) software to acquire size information of the particles.

Next, 5 g of ointment sample is taken and placed in a conical flask, followed by the addition of 30 mL of petroleum ether. The ointment base is completely dissolved by agitation, and then allowed to settle for 15 min. The supernatant is then discarded, and an additional 15 mL of petroleum ether is added, followed by a 5 min settling period, after which the supernatant is removed. Finally, the residue is evaporated to dryness. An appropriate amount of residue is applied to a glass slide, and then a suitable amount of aqueous chloral hydrate is added, followed by drying to achieve transparency. Subsequently, a few drops of 5% diluted glycerol are added, the glass slide is covered, and excess liquid is absorbed using filter paper, resulting in the preparation of an ointment-insoluble plant powder slide.

Additionally, a small amount of powdered medicinal plant material (such as Corydalis Rhizoma and Radix Sanguisorbae Carbonisata) is placed onto a glass slide, and a suitable amount of aqueous chloral hydrate is added, followed by moderate heat treatment to prepare a microscopic slide for plant reference material. It is worth noting that saltpeter and alum are soluble in highly polar solvents, hence liquid paraffin is chosen as the dispersant when preparing slides.

### Statistical analysis

Data statistical analysis was conducted using Origin 2023b (Origin Lab, US). The JMP Pro software (version 17, SAS, Cary, NC) was utilized for stepwise regression model development and determination of parameter significance in the model through analysis of variance (*p*< 0.05). Additionally, JMP Pro was employed used to conduct simulation experiments, decision tree analysis, and Gaussian process analysis.

## Results and discussion

### Rheological properties

The research object of rheology often has dual properties, which include the flow property of liquids and the elastic deformation property of solids [41]. Ointment belongs to semi-solid preparation, which can maintain a certain solid state when standing, and show flow and deformation of fluid when stirred [42]. In the production process of ointment, fully understanding their rheological properties and influencing factors can better optimize the preparation process [43]. In this experiment, the yield stress, low shear viscosity, high shear viscosity, and storage modulus were evaluated. The results of each response limit obtained from the tests were modeled and optimized through stepwise regression analysis. Subsequently, the obtained models were integrated for a comprehensive analysis, in order to obtain an optimal equilibrium solution. After the evaluation of the ointment, initial settings were established for the low shear viscosity (11000 ~ 18000 Pa·s), high shear viscosity (0 ~ 7 Pa·s), storage modulus (200 ~ 1000 Pa), and yield stress (40 ~ 55 Pa).

### Yield stress

**Fig 1:**
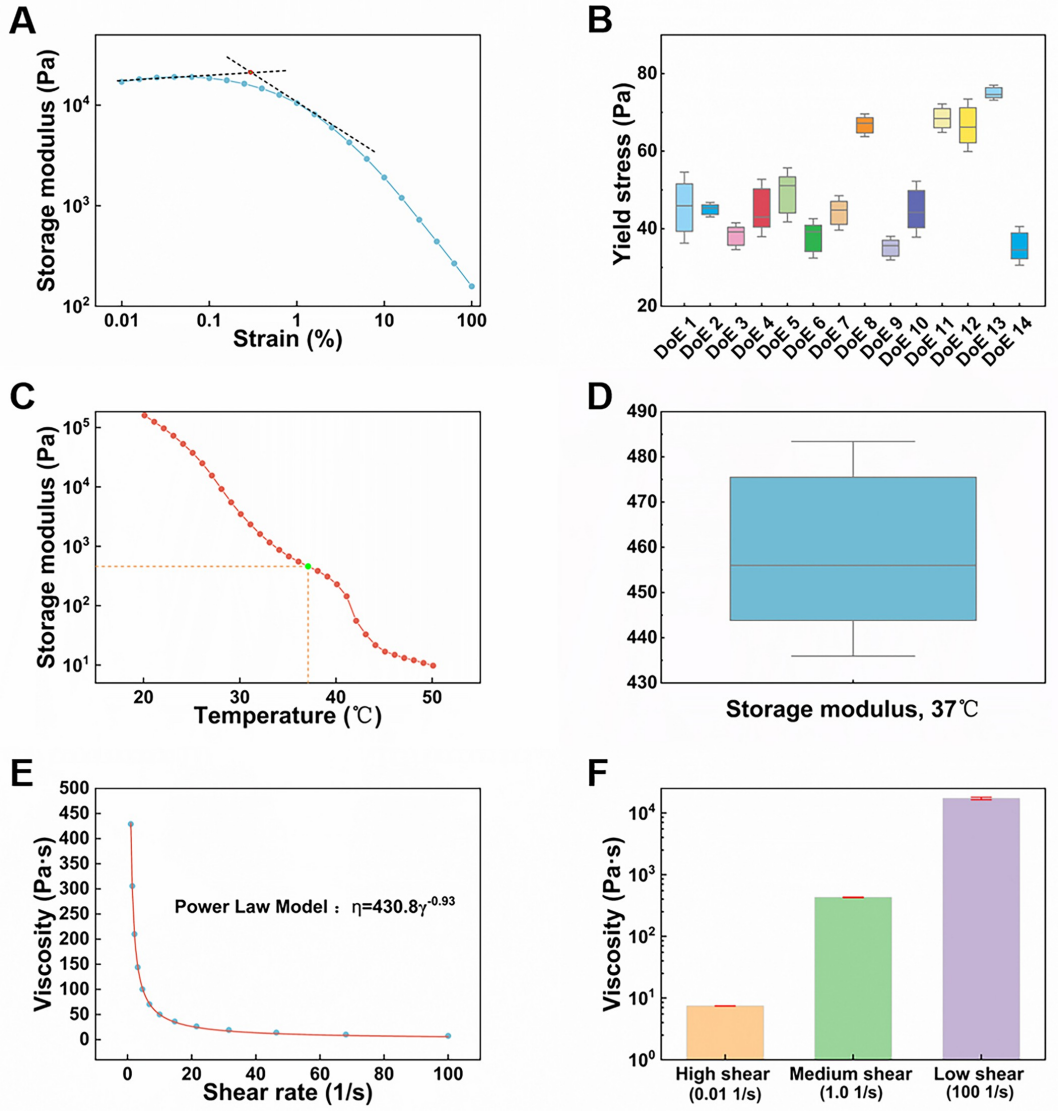
The rheological properties of the ointment. (A) Determine the yield stress starting from the change in storage modulus (DoE 4); (B) Box plot of yield stress for 14 batches of ointment samples; (C) Change in storage modulus under temperature scanning (DoE 4); (D) Box plot of storage modulus at 35°C (DoE 4); (E) Flow Curve—Pseudoplasticity (DoE 4); (F) Paste viscosity at low, medium, and high shear rates (DoE 4).

The yield stress is related to the fluidity of the ointment and reflects the intermolecular force in the microstructure network [41]. The ointment should have enough yield stress to prevent it from flowing out of the container when placed at random [41–43]. At the same time, it should not be too large, otherwise the ointment will not be easy to spread on the skin during use [44,45]. The yield stress values were determined by the variation in the stored energy modulus during strain scans (Fig 1A). Among the 14 batches of ointments, the yield stress values ranged from 35.0 Pa to 75.1 Pa, with an average of 49.4 Pa and a standard deviation of 16.5 Pa (Fig 1B, specific information can be found in S1 Table). DoE8, DoE11, and DooE12 had higher yield stress, requiring higher stress to initiate flow. DoE3 and DoE14 had lower yield stress, making them relatively easier to flow. Both excessively high or low yield stress can impact the actual clinical experience of the ointment.

### Storage modulus

Storage modulus is a physical quantity that describes the elastic properties of materials. The higher the storage modulus, the stronger the ability of the material to restore to its original state after stress, indicating that the material is more rigid [46]. Too high storage modulus will cause the ointment being stiff and difficult to apply evenly on the skin, thus affecting the effective delivery and therapeutic effect of drugs [41]. The change in temperature will directly affect the storage modulus of the ointment. Generally, as the temperature increases, the storage modulus of the ointment will decrease and the ointment to become softer. This is because the rise of temperature will promote the thermal movement of molecules in the ointment, resulting in the weakening of the interaction between molecules, which will reduce the hardness of the ointment [47]. According to the change law of the storage modulus of the ointment at different temperatures, the formula and preparation process of the ointment can be adjusted to achieve the required performance and texture. As shown in the graph, the storage modulus decreases with increasing temperature (Fig 1C). At 37°C, the storage modulus of the ointment ranged from 4463.3 Pa to 226.7 Pa (Fig 1D, specific information can be found in S2 Table). Ointments prepared using different formulations exhibited significant fluctuations at human body temperature. Shanshan W [48] et al. used temperature scanning to determine the phase transition temperature of estradiol biodegradable vaginal thermosensitive in situ gel, ensuring patient compliance and good storage stability during its use in the vaginal cavity. The change in temperature will directly affect the storage modulus of the ointment, and then affect the performance and quality of ointment.

### Shear viscosity

Viscosity is the resistance produced by the relative motion between particles of a fluid under the action of external forces [41]. In the preparation of ointment, viscosity is an important property parameter, which directly affects the fluidity of the ointment. Flow is an irreversible deformation process, and the difficulty of flow is closely related to viscosity [41,42]. The viscosity of the ointment gradually decreases with increasing shear rate, showing shear thinning behavior (Fig 1E and 1F, specific information can be found in S3 Table). This is because long-chain molecules and irregular particles are entangled with each other in the static state, and the apparent viscosity is large; Under the action of shear stress, the molecules will be aligned according to the flow direction, and the resistance and apparent viscosity will be reduced, showing the shear thinning effect. To describe the flow properties of the ointment, we used the flow index to reflect the relationship between shear rate and shear stress, which also serves as an evaluation of its storage and spreadability [49,50]. The results of the power-law model showed that the flow indices of the ointment were all between 0 and 1, indicating that the ointment is a shear-thinning non-Newtonian fluid (Table 4). Apparent viscosity is used to describe the viscosity or viscoelastic properties of a fluid under specific flow conditions. The apparent viscosity values of the ointment ranged from 173.5 to 783 Pa·s, indicating different properties of the ointment under different conditions.

**Table 4:** Apparent viscosity and flow index for all DoE estimate using power law model.

Formulation	Apparent Viscosity (Pa·s)	Flow Index	R^2^
DoE 1	173.5	0.32	0.9975
DoE 2	233.4	0.20	0.9996
DoE 3	332.2	0.14	0.9976
DoE 4	430.8	0.07	0.9989
DoE 5	444.5	0.03	0.9995
DoE 6	728.4	0.05	0.9998
DoE 7	403.2	0.18	0.9996
DoE 8	215.8	0.15	1
DoE 9	372.8	0.18	0.9991
DoE 10	188.2	0.12	0.9992
DoE 11	244.6	0.24	0.9988
DoE 12	783	0.12	0.9903
DoE 13	406.1	0.17	0.9957
DoE 14	177.2	0.06	0.9973

### Fit with stepwise regression analysis

Through stepwise regression analysis, a statistical model was established to estimate the yield stress, storage modulus, low shear viscosity, and high shear viscosity. The overall fit of the model (Table 5), with a goodness of fit (R^2^ > 0.9) and significance (*p* > 0.05), indicated that the model had statistical significance and could provide a scientific description of the relationship between variables.

**Table 5:** Stepwise regression fitting equation.

Response	Equation of fit	R^2^	*p*-value
High shear viscosity(Pa·s)	5.76+2.47×(Drug ratio-35)/15+0.30×(Mixing speed-1050)/750-0.21×(Dosage of petroleum-77.5)/7.5–0.61×(Dosage of liquid paraffin-7.5)/2.5+Match{‘Method A’ = -0.50; ‘Method B’ = 0.50}+1.69×(Drug ratio-35)/15×(Drug ratio-35)/15-0.78×(Mixing speed-1050)/750×(Dosage of liquid paraffin-7.5)/2.5+(Drug ratio-3.5)/15×Match{‘Method A’ = -0.34; ‘Method B’ = 0.34}+(Dosage of petroleum-77.5)/7.5×Match{‘Method A’ = 0.29; ‘Method B’ = -0.29}	0.99473	0.0003
Low shear viscosity(Pa·s)	17855.51+5993.11×(Drug ratio-35)/15-326.77×(Mixing time-25)/20-1507.23×(Mixing speed-1050)/750-998.91×(Dosage of petroleum-77.5)/7.5–1710.11×(Dosage of liquid paraffin-7.5)/2.5+Match{‘Method A’ = -1809.43; ‘Method B’ = 1809.43}-1083.93×(Mixing time-25)/20×(Mixing speed-1050)/750-6274.29×(Mixing time-25)/20×(Dosage of liquid paraffin-7.5)/2.5+(Mixing speed-1050)/750×Match{‘Method A’ = -1668.13; ‘Method B’ = 1668.13}	0.95905	0.0188
Storage modulus(Pa)	550.40+1307.12×(Drug ratio-35)/15-283.03×(Mixing time-25)/20+104.60×(Mixing speed-1050)/750-125.86×(Dosage of petroleum-77.5)/7.5–211.11×(Dosage of liquid paraffin-7.5)/2.5+Match{‘Method A’ = -338.31; ‘Method B’ = 338.31}+(Drug ratio-35)/15×(Drug ratio-35)/15×942.47+(Drug ratio-35)/15×Match{‘Method A’ = -563.69; ‘Method B’ = 563.69}+(Mixing time-25)/20×Match{‘Method A’ = 215.64; ‘Method B’ = -215.64}	0.98888	0.0015
Yield stress(Pa)	42.00+2.23×(Mixing time-25)/20-3.39×(Mixing speed-1050)/750+8.83×(Dosage of petroleum-77.5)/7.5–6.96×(Dosage of liquid paraffin-7.5)/2.5+Match{‘Method A’ = -2.33; ‘Method B’ = 2.33}+(Mixing speed-1050)/750×(Mixing speed-1050)/750×10.86+(Mixing time-25)/20×(Dosage of liquid paraffin-7.5)/2.5×-5.04+(Dosage of petroleum-77.5)/7.5×(Dosage of liquid paraffin-7.5)/2.5×-7.37	0.913938	0.0260

**Fig 2:**
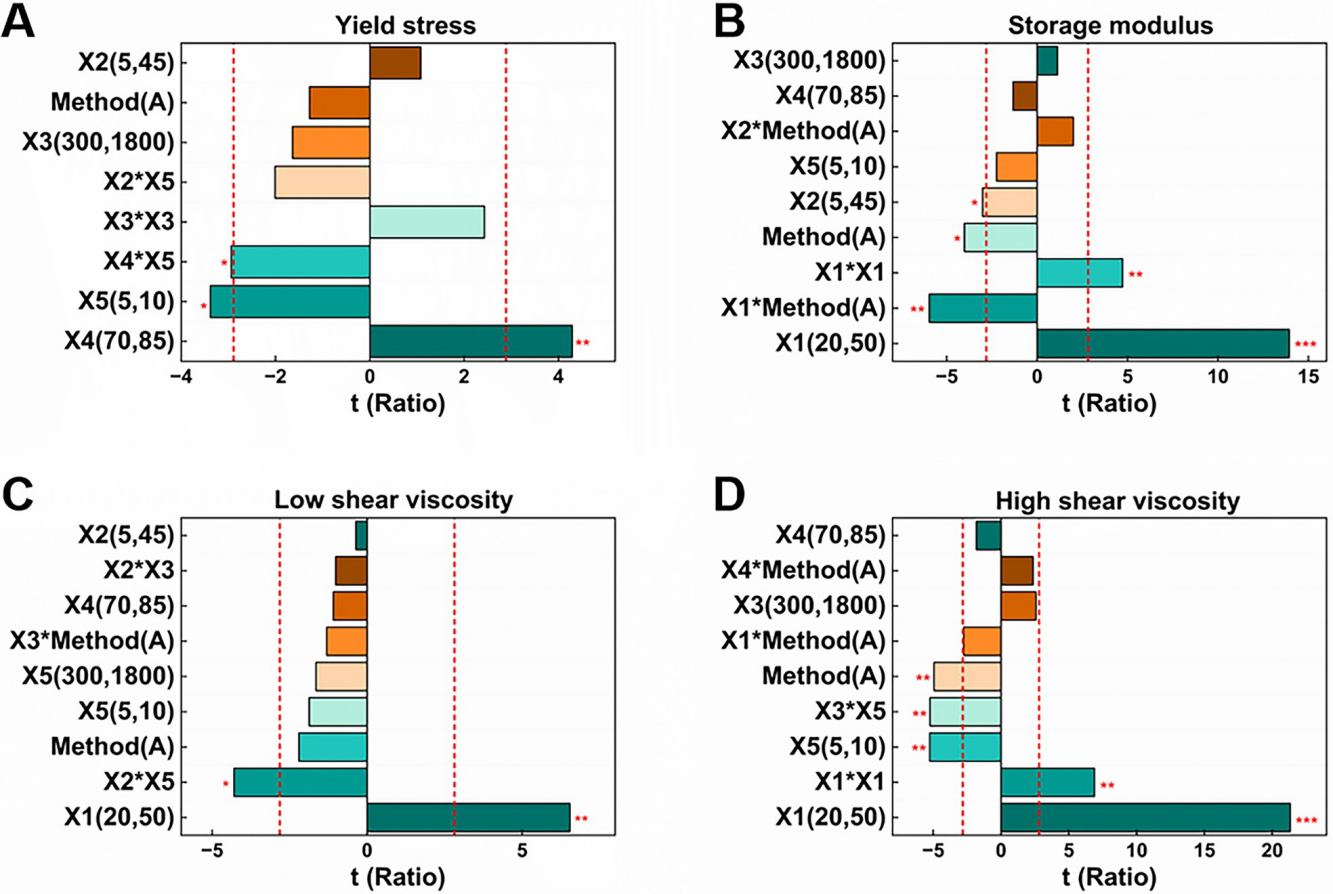
Sorted parameter estimates based on t-Ratio for various formulation and process parameters. (A)Yield stress; (B)Storage modulus; (C)High shear viscosity; (D) Low shear viscosity. *p<0.05, **p<0.01, ***p<0.001.

To identify the variables that significantly influenced the response, the estimated t-ratios of the formulation and process parameters were analyzed statistically (Figs 2 and S2). The ratio of petroleum to liquid paraffin had a significant impact on the yield stress, indicating that adjusting the consistency of the ointment base through the proportion of petroleum and liquid paraffin might influence the dispersion of other components in the material. There was also a significant interaction between the two variables, potentially causing changes in the internal structure of the ointment, thereby affecting the yield stress. The research by PENA et al. [51,52] also showed that the ointment base, such as white petrolatum and mineral oil, significantly affected the structure of the ointment, with petrolatum contributing to its stability through a three-dimensional network structure. The drug loading had a highly significant effect on the storage modulus, high shear viscosity, and low shear viscosity. Increasing drug loading usually means introducing more solid components, thereby altering the physical and rheological properties of the ointment. The addition of drugs most directly affects the viscosity of the ointment, potentially weakening its flow phase and increasing viscosity, while also lead to a higher storage modulus, making the ointment more rigid. The negative correlation between drug loading and storage modulus is consistent with the findings of XU [53]. Liquid paraffin demonstrated a significant viscosity effect under high shear conditions, while its influence under low shear conditions was not significant. This could be attributed to the use of liquid paraffin to adjust the viscosity of the ointment, making it into a shear-thinning non-Newtonian fluid. Under high shear rates, liquid paraffin gradually thins, causing the destruction of the ointment structure and reducing friction between molecules or particles, resulting in a noticeable thinning effect. Under low shear rates, the internal structure of the ointment remains relatively stable, leading to higher viscosity and making it difficult for the thinning effect of liquid paraffin to be observed. The method of drug addition during ointment preparation has an important influence on the properties and performance of the ointment. Adding drugs to the molten state of the ointment base at high temperatures may cause drug decomposition or volatilization due to heat sensitivity, thereby affecting the flowability and viscosity of the ointment base. Adding drugs to the ointment base after shaping can avoid drug exposure to high temperatures, helping to preserve the stability of the drug.

**Fig 3:**
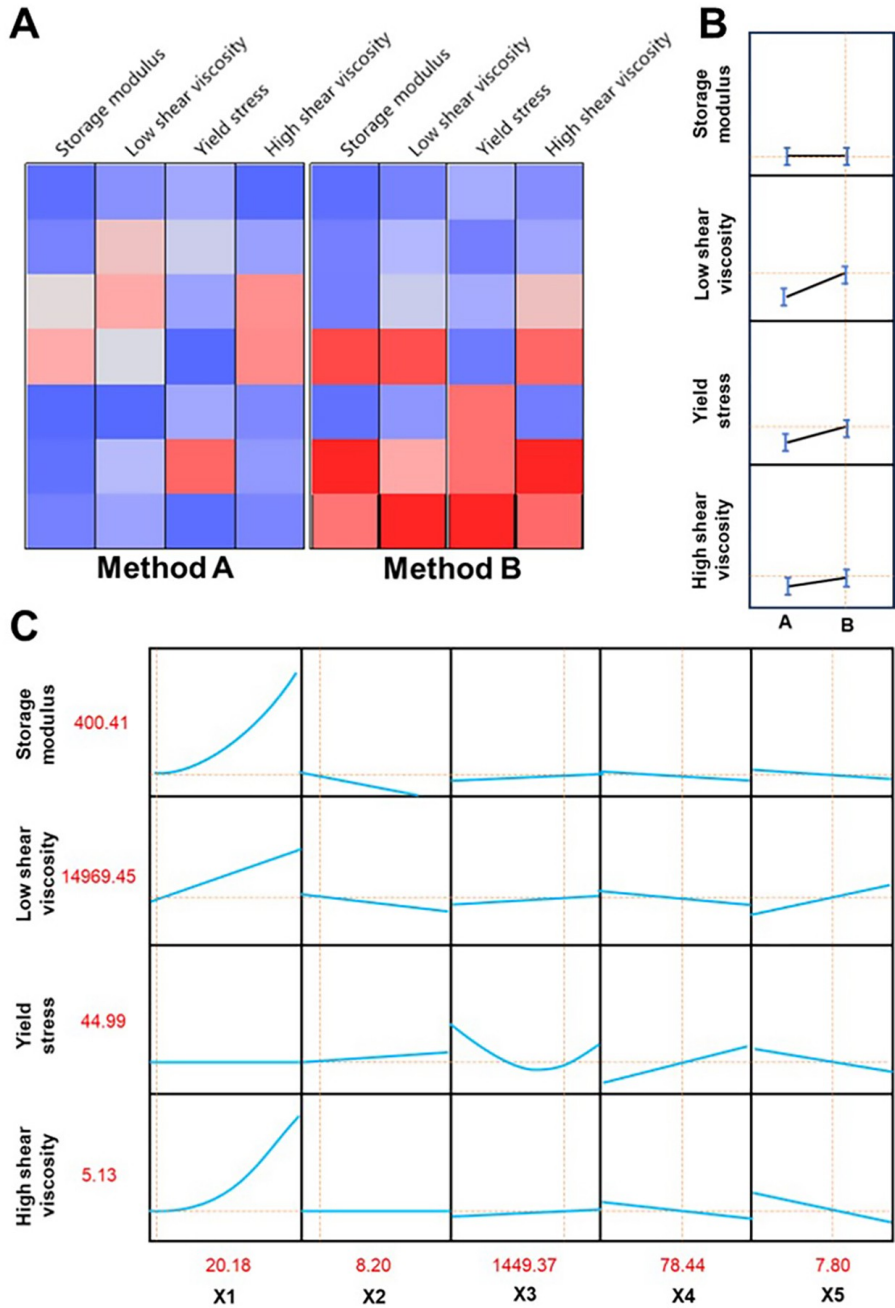
Summary of forecast results. (A) The graphical representation of the methodology on a cell plot; (B) Optimization method; (C) The projected outcomes of the factor prediction.

The cell plot (Fig 3A) of methods A and B indicates that method B has a wider parameter range and is more likely to approach the ideal value. Predictions also show that most parameters tend to favor method B, making it superior to method A (Fig 3B). The predictive profiler reveals that the influence of X1 on storage modulus and high shear viscosity, as well as the effect of X3 on yield stress, demonstrate non-linear relationships. This indicates that changes in these relevant factors can result in relatively significant fluctuations. By maximizing willingness (Fig 3C), we have obtained the optimal equilibrium solution at X1 = 20.18, X2 = 8.20, X3 = 1449.37, X4 = 78.44, and X5 = 7.80. To test its stability, we conducted Monte Carlo simulations as experimental simulations. Monte Carlo simulation is a method that uses random numbers and probability statistics for simulation and analysis. It involves generating a large number of random samples and running simulations to obtain experimental results, followed by statistical analysis of the output values to determine the probability distribution [54,55]. In the experiment, we integrated the models of four response limits and performed 100,000 simulation experiments. The results showed that the defect probability of the ointment preparation was 46.65% (Table 6). A defect rate of nearly half is undesirable in our production process.

**Table 6:** Defect rate at different analysis stages.

Method Defect	Before optimization	After optimization
High shear viscosity	0.02	0.03
Low shear viscosity	0.25	0.05
Storage modulus	0.10	0.03
Yield stress	0.18	0.00006
Overall defect rate	0.46	0.11

### Decision tree optimization

**Fig 4:**
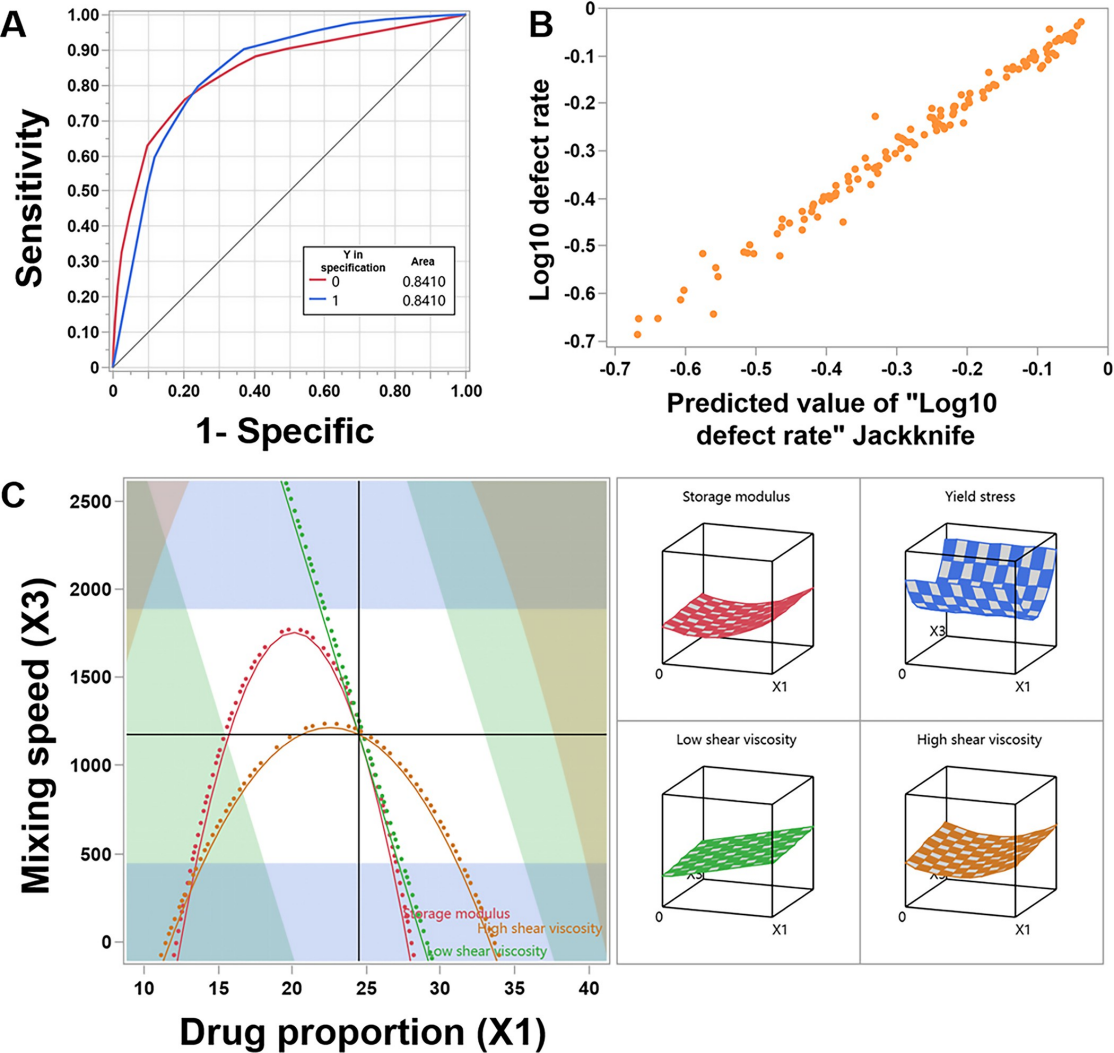
Design optimization. (A) ROC curve based on decision tree model; (B) The residual between the predicted value generated by the Gaussian process model and the actual value; (C) Contour map of predicted results.

**Fig 5:**
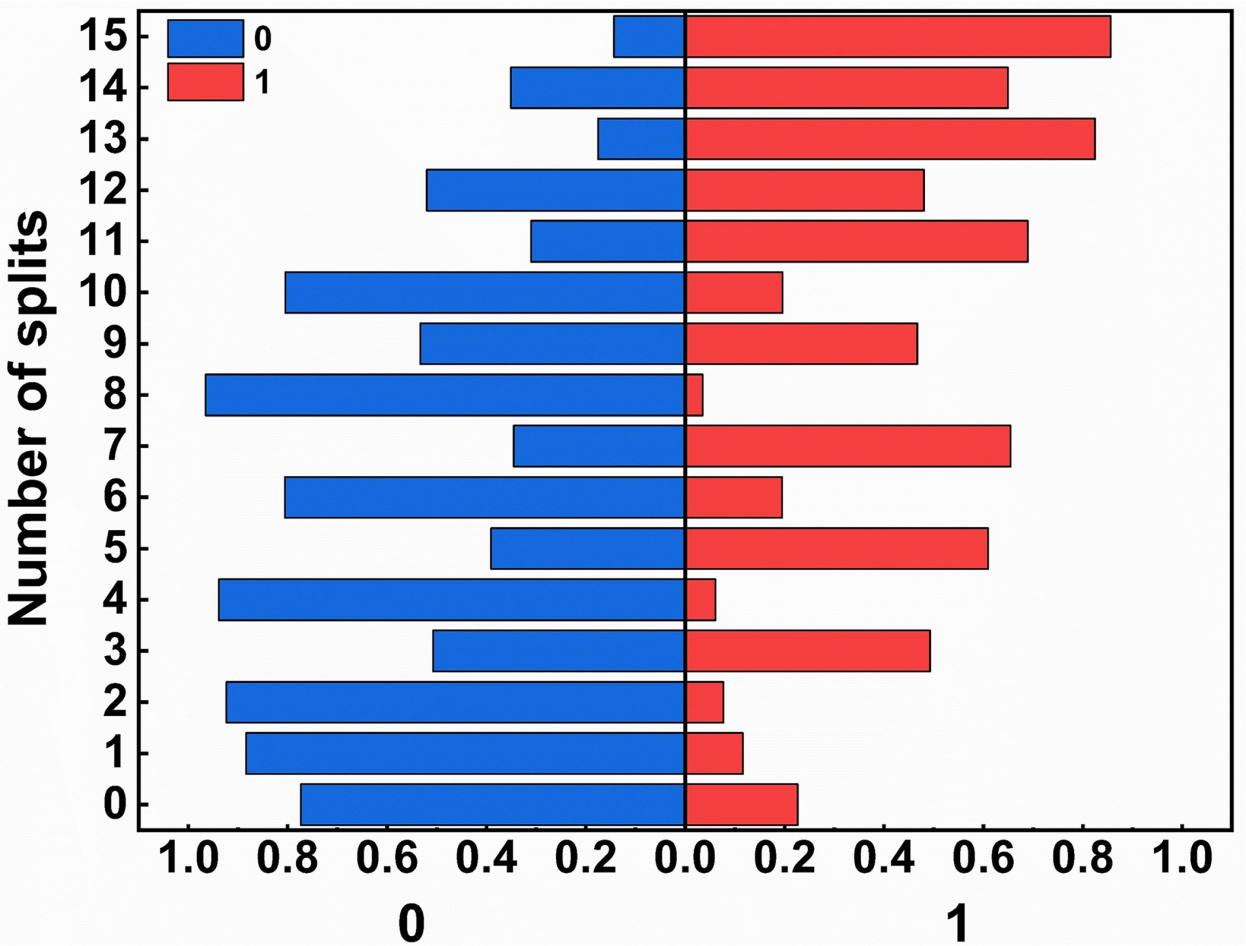
Split results based on decision tree.

To reduce the defect rate, we performed decision tree analysis and developed a model that links the experimental factors with the output being within specification limits. The Receiver Operating Characteristic (ROC) curve (Fig 4A) showed that the area under the curve for the model was 0.8410, exceeding 0.5, indicating good diagnostic capability of the model. By performing 15 decision tree splits (Fig 5), we obtained a defect rate of 14.38%. Further splits were halted as excessively stringent experimental conditions could lead to a decrease in the operational space available in practical settings. Therefore, the chosen range of factors is: X1 = 19.72±4.06, X2 = 5.04±6.42, X3 = 1434.99±267.81, X4 = 78.26±1.76, X5 = 7.41±0.47. The fluctuations of these factors within this range have a negligible impact on the final expected outcome, avoiding drastic fluctuations in rheological characteristics and ensuring the robustness of the design. The design space provides flexibility in adjusting parameters during actual operational processes, thereby preventing the drawbacks of extreme parameter values that may result in decreased product stability. It serves as a design for robustness in parameter settings [56].

### Gaussian process

The Gaussian process is a probability model that models continuous random variables’ distributions. It can be used for optimizing experimental designs, seeking the optimal conditions of experiments through Gaussian process within the overall range [57]. To simulate, Monte Carlo simulations were performed with 100,000 runs in each of the 128 experiments (S4 Table), and a Gaussian fit was applied to the “Log_10_defect rate” obtained in each run. Fig 4B depicts that the points show a linear distribution along the diagonal, indicating that the model possesses good predictive ability. The prediction results showed that the response of the ointment prepared by method B was Y1 = 44.98, Y2 = 5.32, Y3 = 14608, Y4 = 530.7 when the factors X1 = 24.498709, X2 = 7.769771, X3 = 1175.417, X4 = 78.855153, X5 = 6.7251419. At this time, the defect rate of the ointment is the lowest, and the preparation conditions are relatively optimal. For practical convenience, approximated values were taken as X1 = 24.5, X2 = 8, X3 = 1175, X4 = 79, and X5 = 6.7.

The optimized design space of the ointment after decision tree optimization is depicted in Fig 4C, where the white region indicates that the product meets the requirements. Within this design space, a trade-off and exploration among experimental factors are typically necessary to identify the most suitable solution. Following Gaussian fitting, the intersection of the two solid black lines represents the optimal process point with a defect rate of 10.42%. To assess whether our model can accurately predict the influence of all studied parameters on the ointment’s state, three validation experiments were conducted under optimal process conditions (Table 7). The relative standard deviations (RSD) between these three batches ranged from 2.69% to 9.41%, while the RSD with respect to target values ranged from 2.34% to 7.71%. It is generally acceptable for rheometers’ measurements to exhibit fluctuations within a range of 20%. Therefore, considering that our results fall within this specified range, it confirms the effectiveness of our model.

**Table 7:** Summary of verification batches produced at optimal settings.

Output parameter	Target	Validation(*n* = 3)	RSD between validation batches (%)	Deviation of average from target (%)
High shear viscosity	5.32	5.73±0.18	3.17%	7.71%
Low shear viscosity	14608	14266.67±665.83	4.67%	-2.34%
Storage modulus	530.7	559±52.6	9.41%	5.33%
Yield stress	44.98	47.73±1.29	2.69%	6.11%

### Preliminary stability assessment

Upon initial extraction, the ointment exhibits a slight liquefied state in high-temperature environments, but returns to its original consistency when cooled to room temperature. In low-temperature environments, the ointment slightly hardens upon extraction but can be it easily spread on the skin with the appropriate amount. Upon returning to room temperature, no significant changes are observed.

After centrifugation, the ointment does not show any signs of sedimentation. The ointment obtained from the upper, middle, and lower portions of the centrifuge tube exhibits a uniform texture without noticeable granularity.

After a 2-month storage period at room temperature, no signs of deterioration or evident changes in texture were observed. The ointment demonstrates favorable performance in the preliminary assessment of stability.

### Micro analysis

**Fig 6:**
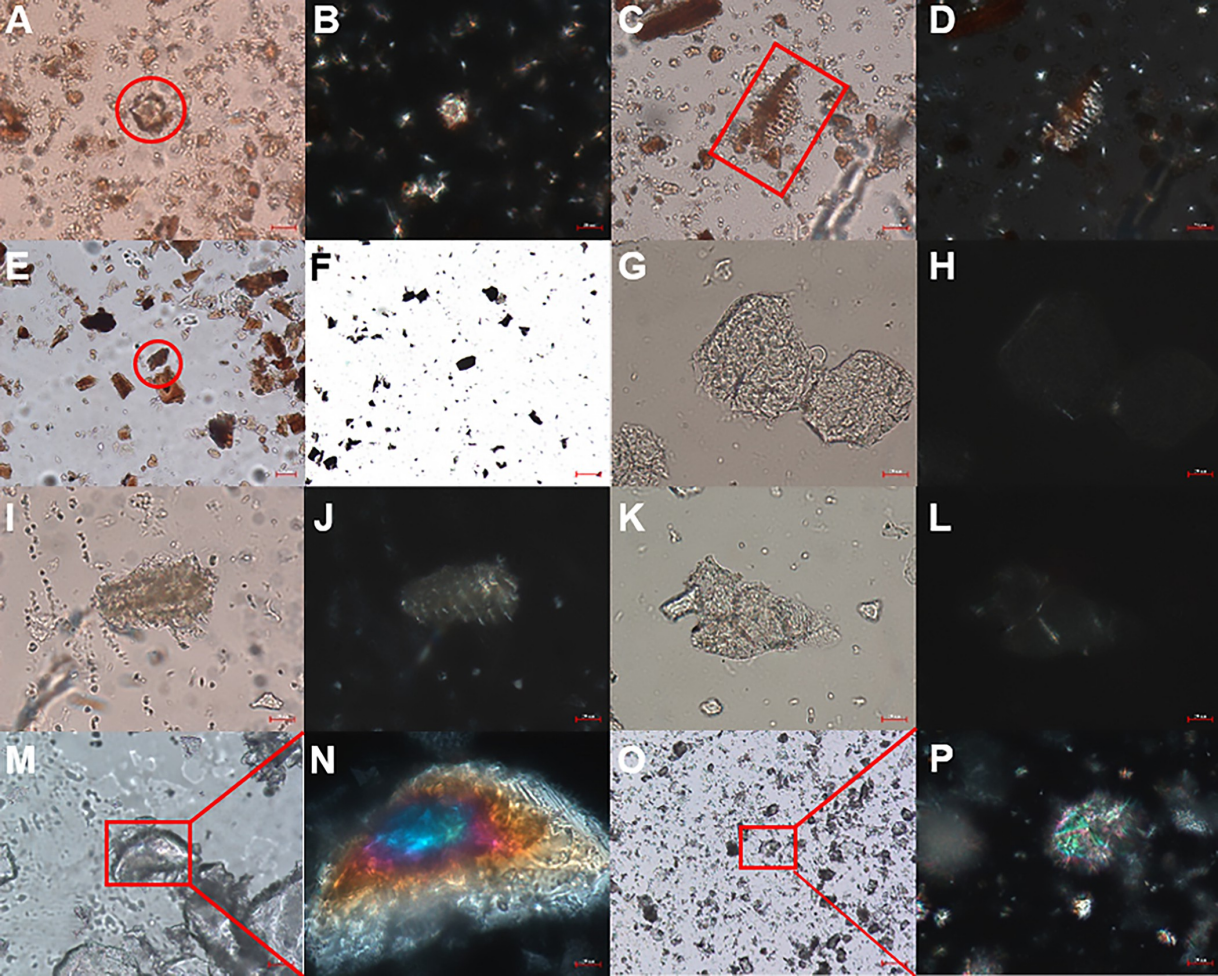
Microscopic analysis of active pharmaceutical ingredients. (A) Radix Sanguisorbae Carbonisata calcium oxalate cluster; (B) Radix Sanguisorbae Carbonisata calcium oxalate cluster (Crossed polars); (C) Radix Sanguisorbae Carbonisata reticum reticulate vessel; (D) Radix Sanguisorbae Carbonisata reticum reticulate vessel (Crossed polars); (E) Radix Sanguisorbae Carbonisata cork cell; (F) Plant soot charcoal particles; (G) Corydalis Rhizoma parenchyma cell; (H) Corydalis Rhizoma parenchyma cell (Crossed polars); (I) Corydalis Rhizoma spiral vessel; (J) Corydalis Rhizoma spiral vessel (Crossed polars); (K) Corydalis Rhizoma sclerenchyma cell;(L)Corydalis Rhizoma sclerenchyma cell(Crossed polars); (M) Natrii Sulfas; (N) Natrii Sulfas (Crossed polars); (O) Calcine Alunite;(P) Calcine Alunite (Crossed polars). (The scale of F, M and O is 100 μm, and the rest are 20 μm).

In Fig 6, the microscopic characteristics of the Radix Sanguisorbae Carbonisata powder include clusters of calcium oxalate crystals, reticulate vessels, and cork cells. Under the observation of a polarizing microscope, it can be observed that calcium oxalate crystals and reticulate vessels exhibit distinct polarization effects, while cork tissues do not display any polarization phenomenon. The Corydalis Rhizoma powder had parenchyma cell, spiral vessel and sclerenchyma cell, in which parenchyma cell were filled with pasted granule, and the walls of sclerenchyma cells were thickened in a beaded way. The spiral vessel showed significant polarization interference effect, and the edges of sclerenchyma cells and parenchyma cells also showed slight polarization interference effect. Regarding the plant soot, we are able to discern the noticeable presence of charcoal particles. The constituents of Natrii Sulfas and Calcine Alunite are Na_2_SO_4_·10H_2_O and KAl(SO_4_)_2_ respectively, exhibiting vibrant colors under the polarizing microscope, making them easily identifiable.

**Fig 7:**
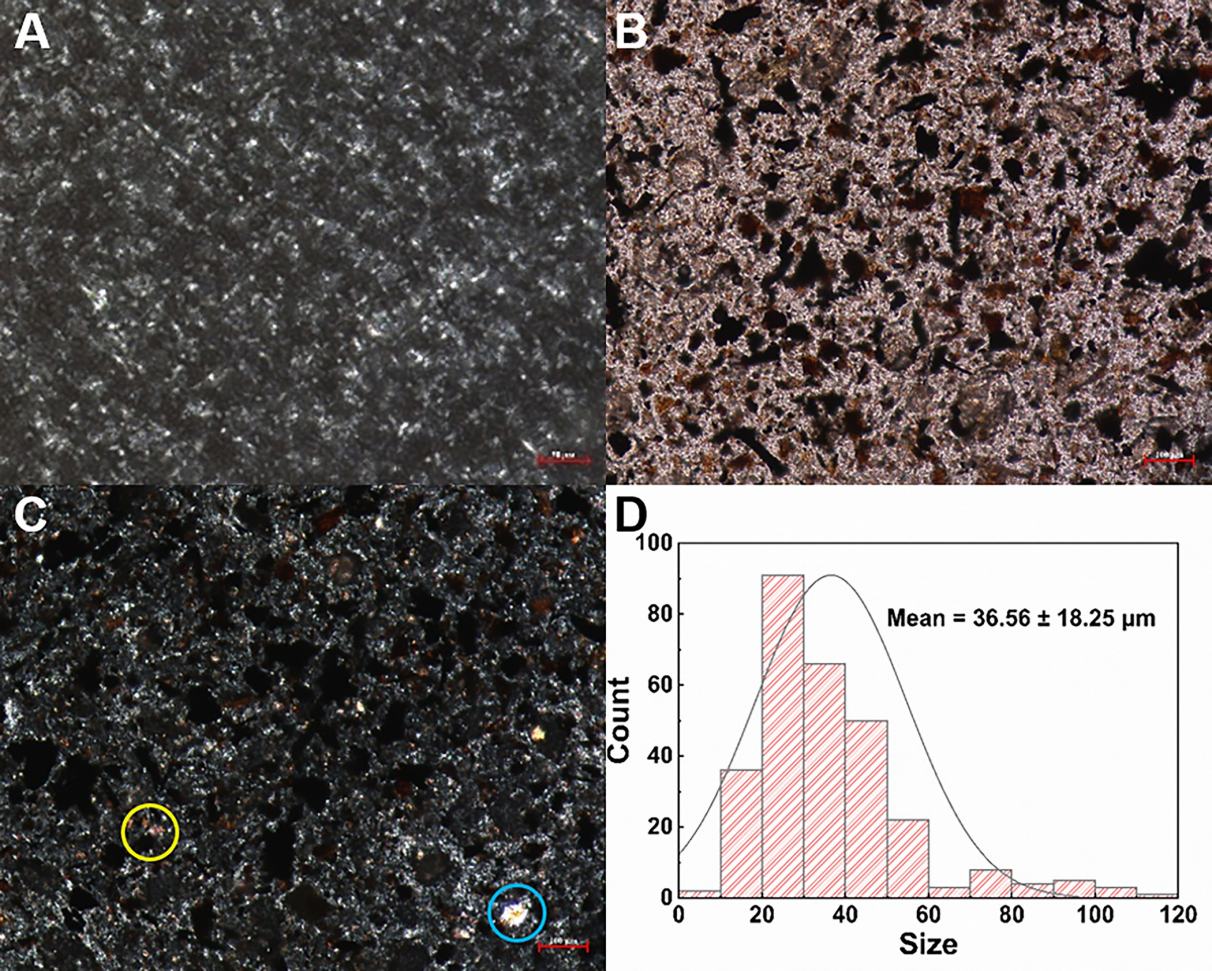
Microscopic analysis of ointment. (A) Ointment base (Crossed polars); (B) The ointment shows microscopic characteristics; (C) Microscopic characteristics of ointment (Crossed polars); (D) Particle size analysis. (The scale is 100 μm).

Upon examining Fig 7, it is evident that the distribution of the powdered medication within the ointment base is relatively homogeneous, and no agglomeration phenomenon was observed. The bright areas in the ointment base are a result of the refractive index of the ointment base materials, such as petrolatum and lanolin [48]. The particle sizes were analyzed and measured using Image J software, with an average particle size of approximately 36.56 μm and no particles larger than 180 μm were found. The distinct blue and red circles represent Natrii Sulfas and Calcine Alunite, respectively, allowing for easy identification. Upon removal of the ointment base, the internal characteristics of the powdered medication can be observed (additional data shown in S3 Fig), which corroborates the relevant images in Fig 4.

## Conclusion

The DSD effectively avoids the cross-interaction of second-order and lower-order effects by establishing a supersaturated model. After stepwise regression fitting and modeling analysis, it was found that the drug loading and ointment base ratio significantly influence the rheological properties of the ointment, serving as important factors affecting the rheological characteristics of the ointment. Therefore, it is necessary to control the value range of relevant parameters to obtain the desired product.

The determination of these parameters value ranges involves a classification problem, which can be efficiently solved using decision trees. By splitting the decision tree, the design space of the ointment can be obtained, avoiding extreme values in traditional experimental design parameter selection and providing a more robust choice of parameter values.

Lastly, with the assistance of Gaussian processes, optimal parameters can be obtained across the entire range. This not only optimizes the design but also provides further tolerance space, ensuring a more stable production of the ointment. The results showed that the drug proportion was 24.5%, mixing time was 8 min, mixing speed was 1175 rpm, petrolatum dosage was 79 g, and liquid paraffin dosage was 6.7 g. Method B was used to prepare the WYH ointment, and the verification results met expectations. In summary, these processes provide a robust formulation process for the ointment.

In the past, simple trial-and-error method and single factor experimental design were often used to optimize the preparation process of traditional Chinese medicine ointment, which were often inefficient and the results were not reliable enough. In contrast, the systematic method can consider the interaction between various factors more comprehensively, and carry out targeted optimization for each key parameter, so as to find the best combination of preparation parameters. This personalized optimization method can better meet the specific needs of the ointment preparation process and improve the efficiency and accuracy of optimization.

However, it is important to note that DSD have their inherent limitations. They generally do not allow for a separate estimate of the secondary effects of each factor, which can introduce ambiguity into the analysis. In addition, it is worth considering that deterministic screening designs may not fully capture the complexity of higher-order interactions between factors.

## Supporting information

S1 Fig Correlation chromatic graph of complete quadratic model. (DOCX)

S2 Fig Factor—response interaction. (DOCX)

S3 Fig Analysis of plant powder in ointment. (DOCX)

S1 Table Yield stress data of DoE formula. (DOCX)

S2 Table Storage modulus (Pa) and temperature (°C) data of DoE formula. (DOCX)

S3 Table Viscosity (Pa.s) and shear rate (s-1) data of DoE formula. (DOCX)

S4 Table Summary of 128 simulation experiments. (DOCX)
