# Supplementary figures and images for: Refining the rheological characteristics of high drug loading ointment via SDS and machine learning
Source: PLoS One. 2024 May 9;19(5):e0303199. doi: 10.1371/journal.pone.0303199 (PMC11081290; doi:10.1371/journal.pone.0303199)

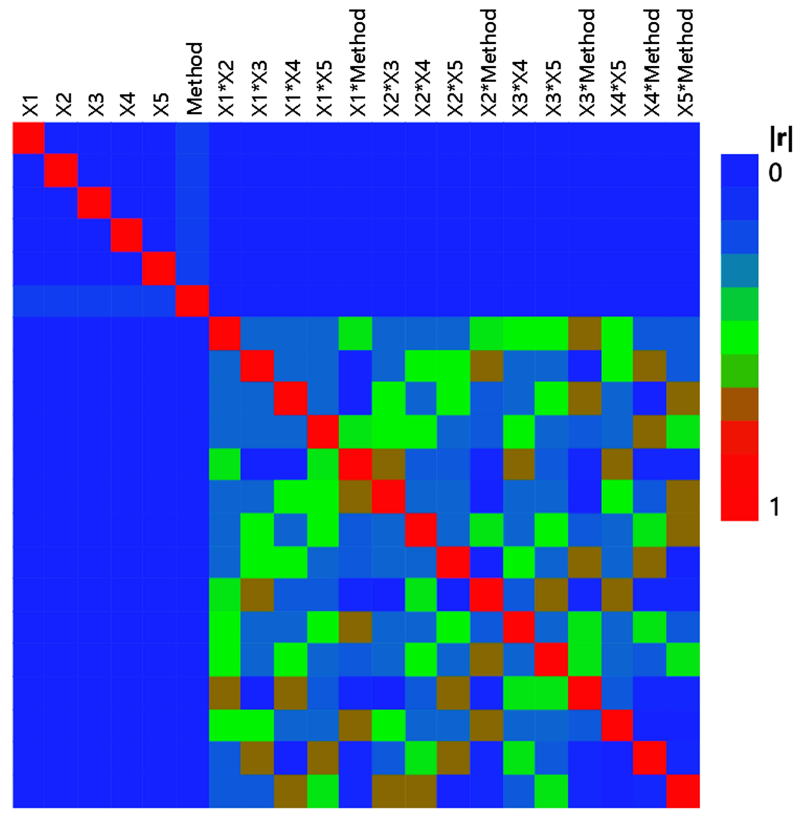


**S1 Fig. Correlation chromatic graph of complete quadratic model**

Supplement: S1 Fig — (DOCX) [file pone.0303199.s001.docx]
