# Supplementary material for: Refining the rheological characteristics of high drug loading ointment via SDS and machine learning
Source: PLoS One. 2024 May 9;19(5):e0303199. doi: 10.1371/journal.pone.0303199 (PMC11081290; doi:10.1371/journal.pone.0303199)
Supplement: S2 Fig — (DOCX) [file pone.0303199.s002.docx]

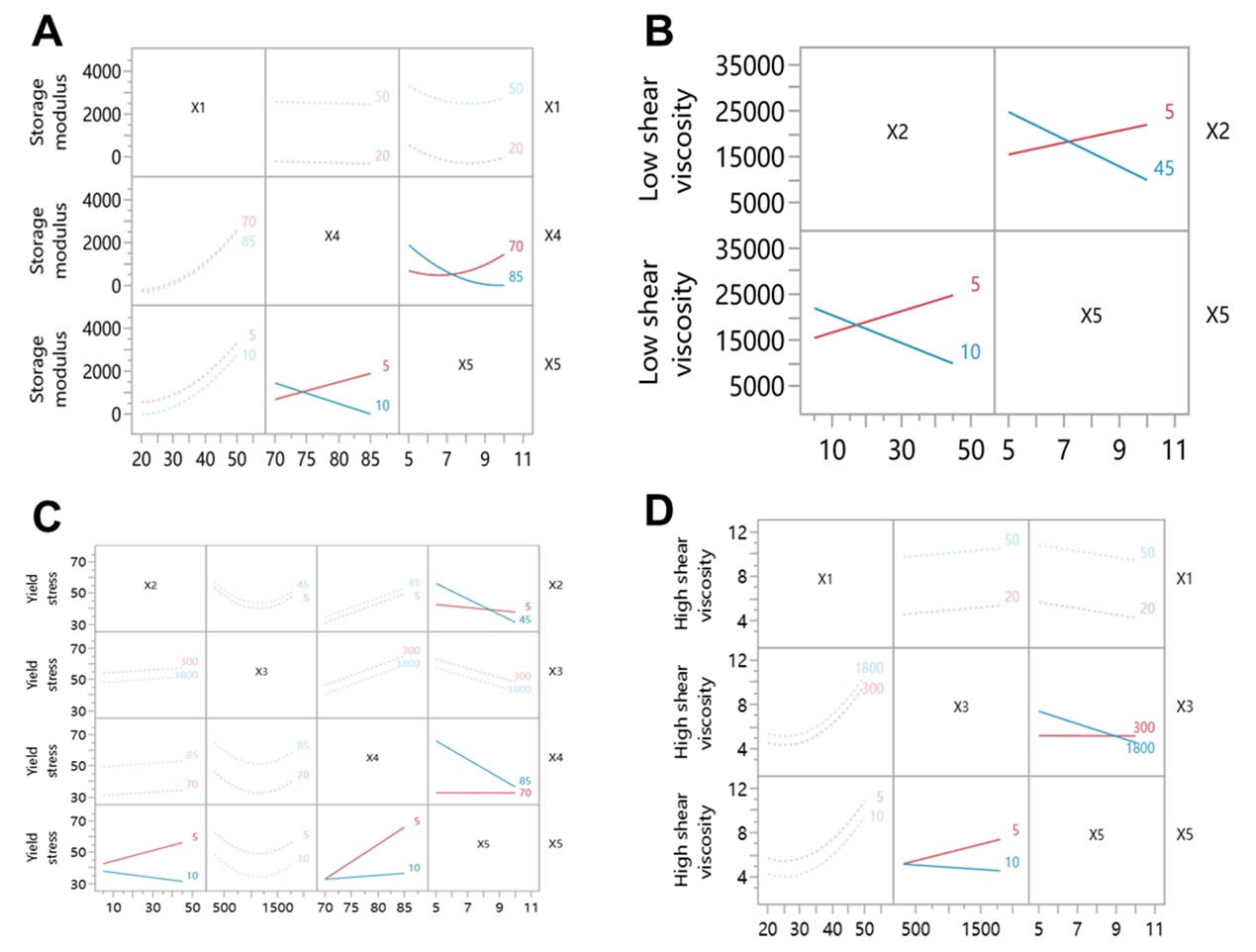


**S1 Fig. Factor - response interaction** (A) storage modulus, (B) low shear viscosity, (C) yield stress, (D) high shear viscosity
