# Supplementary material for: Refining the rheological characteristics of high drug loading ointment via SDS and machine learning
Source: PLoS One. 2024 May 9;19(5):e0303199. doi: 10.1371/journal.pone.0303199 (PMC11081290; doi:10.1371/journal.pone.0303199)
Supplement: S3 Fig — (DOCX) [file pone.0303199.s003.docx]

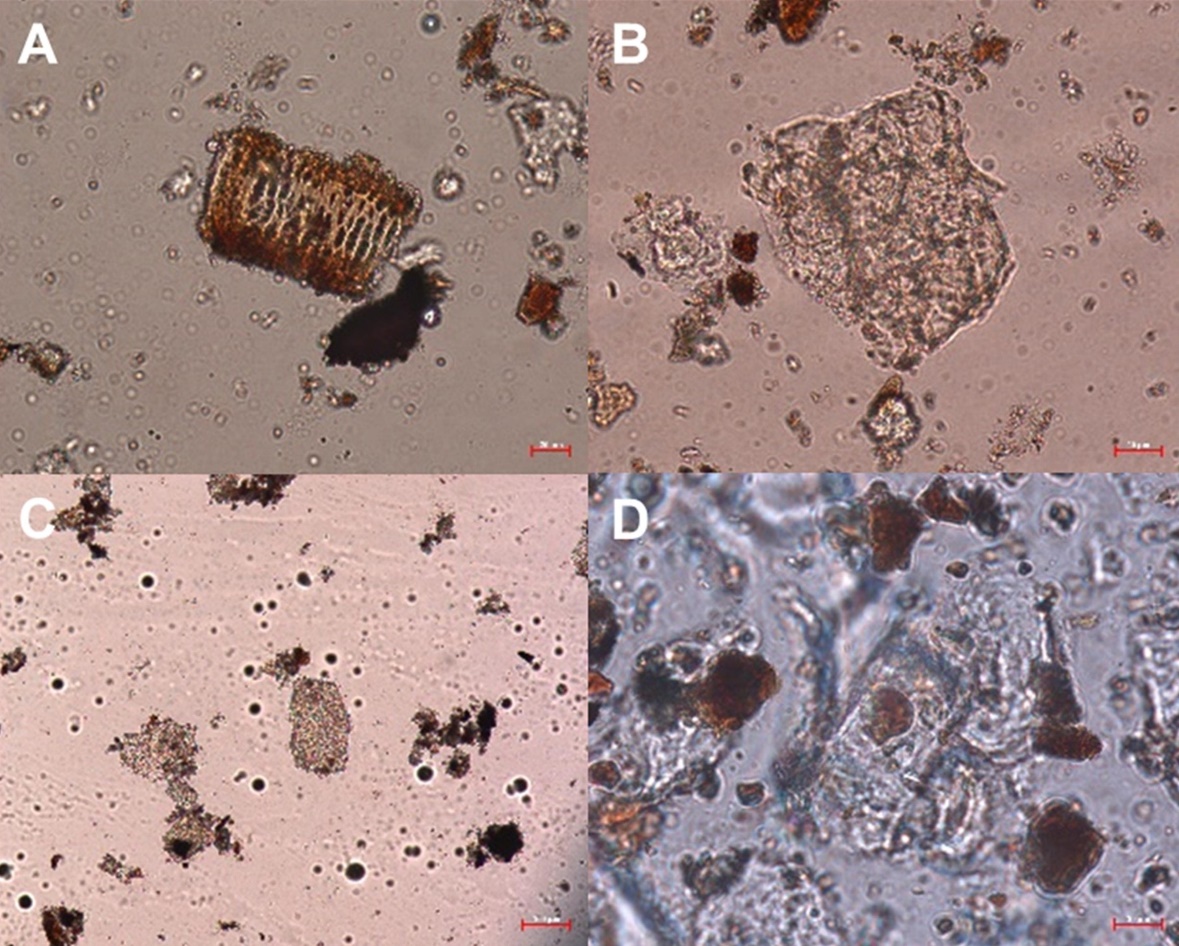


**S1 Fig. Analysis of Plant Powder in Ointment.** (A) Vessel; (B) Sclerenchyma cell; (C) Parenchyma cell; (D) Cork cell. (The scale is 20 μm)
