# Supplementary material for: Refining the rheological characteristics of high drug loading ointment via SDS and machine learning
Source: PLoS One. 2024 May 9;19(5):e0303199. doi: 10.1371/journal.pone.0303199 (PMC11081290; doi:10.1371/journal.pone.0303199)
Supplement: S1 Table — (DOCX) [file pone.0303199.s004.docx]

**S1 Table. Yield stress data of DoE formula (*n*=3)**

| Formulation | Yield stress (Pa) | |
| --- | --- | --- |
|  | Mean | SD |
| DOE-1 | 45.4 | 6.1 |
| DOE-2 | 44.9 | 1.2 |
| DOE-3 | 38.1 | 2.3 |
| DOE-4 | 45.3 | 4.9 |
| DOE-5 | 48.7 | 4.6 |
| DOE-6 | 37.5 | 3.4 |
| DOE-7 | 44.1 | 3.0 |
| DOE-8 | 66.7 | 2.0 |
| DOE-9 | 35.0 | 2.0 |
| DOE-10 | 45.0 | 4.8 |
| DOE-11 | 68.5 | 2.5 |
| DOE-12 | 66.7 | 4.5 |
| DOE-13 | 75.1 | 1.3 |
| DOE-14 | 35.6 | 3.3 |
