# Supplementary material for: Refining the rheological characteristics of high drug loading ointment via SDS and machine learning
Source: PLoS One. 2024 May 9;19(5):e0303199. doi: 10.1371/journal.pone.0303199 (PMC11081290; doi:10.1371/journal.pone.0303199)
Supplement: S2 Table — (DOCX) [file pone.0303199.s005.docx]

**S2 Table.**  **Storage modulus (Pa) and temperature (℃) data of DoE formula (*n*=3)**

| **Temperature**  **[°C]** | **DoE 1** | | **DoE 2** | | **DoE 3** | | **DoE 4** | | **DoE 5** | | **DoE 6** | | **DoE 7** | | **DoE 8** | | **DoE 9** | | **DoE 10** | | **DoE 11** | | **DoE 12** | | **DoE 13** | | **DoE 14** | |
| --- | --- | --- | --- | --- | --- | --- | --- | --- | --- | --- | --- | --- | --- | --- | --- | --- | --- | --- | --- | --- | --- | --- | --- | --- | --- | --- | --- | --- |
|  | **Mean** | **SD** | **Mean** | **SD** | **Mean** | **SD** | **Mean** | **SD** | **Mean** | **SD** | **Mean** | **SD** | **Mean** | **SD** | **Mean** | **SD** | **Mean** | **SD** | **Mean** | **SD** | **Mean** | **SD** | **Mean** | **SD** | **Mean** | **SD** | **Mean** | **SD** |
| **20.1** | **70233.3** | **17931.6** | **77066.7** | **5229.1** | **66333.3** | **30022.2** | **159666.7** | **15567.1** | **63800.0** | **2500.0** | **160666.7** | **10016.7** | **157666.7** | **5033.2** | **47733.3** | **4737.4** | **56966.7** | **10084.8** | **45633.3** | **1514.4** | **71833.3** | **57.7** | **316333.3** | **19655.4** | **258333.3** | **14640.1** | **60166.7** | **33316.3** |
| **21.1** | **60233.3** | **15662.2** | **64300.0** | **4132.8** | **55833.3** | **24298.2** | **125000.0** | **13527.7** | **58766.7** | **1850.0** | **133666.7** | **9291.6** | **131666.7** | **3511.9** | **40766.7** | **2844.9** | **46533.3** | **5179.1** | **38600.0** | **1228.8** | **59966.7** | **929.2** | **266333.3** | **16289.1** | **213666.7** | **12583.1** | **51733.3** | **27078.8** |
| **22.1** | **50866.7** | **13416.5** | **53300.0** | **2954.7** | **47566.7** | **19107.2** | **96700.0** | **8926.9** | **51200.0** | **2300.0** | **111333.3** | **8736.9** | **109000.0** | **2000.0** | **33766.7** | **2409.0** | **39866.7** | **5320.1** | **33600.0** | **1153.3** | **51333.3** | **776.7** | **221333.3** | **13051.2** | **173000.0** | **11000.0** | **44933.3** | **19328.3** |
| **23.1** | **43033.3** | **10200.2** | **43500.0** | **1852.0** | **41000.0** | **13923.7** | **72600.0** | **6458.3** | **45366.7** | **1950.0** | **90266.7** | **8071.1** | **88200.0** | **953.9** | **30800.0** | **1479.9** | **36833.3** | **461.9** | **30133.3** | **1724.3** | **44166.7** | **305.5** | **182000.0** | **10148.9** | **136000.0** | **10535.7** | **39300.0** | **12157.7** |
| **24.1** | **35000.0** | **7854.3** | **35033.3** | **850.5** | **35633.3** | **9053.4** | **53000.0** | **4454.2** | **37700.0** | **1900.0** | **71933.3** | **7558.0** | **70000.0** | **360.6** | **26966.7** | **929.2** | **33800.0** | **2165.6** | **28200.0** | **1493.3** | **37400.0** | **100.0** | **146333.3** | **7023.8** | **103966.7** | **9219.7** | **33733.3** | **6562.3** |
| **25.1** | **27333.3** | **5707.3** | **26800.0** | **346.4** | **28600.0** | **5866.0** | **37400.0** | **3109.7** | **28900.0** | **1800.0** | **56066.7** | **7392.1** | **54100.0** | **1705.9** | **22433.3** | **550.8** | **29733.3** | **513.2** | **23133.3** | **665.8** | **30633.3** | **57.7** | **114333.3** | **4618.8** | **76266.7** | **7167.5** | **27200.0** | **3204.7** |
| **26.1** | **20533.3** | **4148.9** | **19800.0** | **173.2** | **21933.3** | **3876.0** | **24933.3** | **2052.6** | **22300.0** | **900.0** | **42300.0** | **7458.6** | **40000.0** | **2260.5** | **18333.3** | **57.7** | **23866.7** | **907.4** | **16500.0** | **264.6** | **23500.0** | **100.0** | **85766.7** | **3412.2** | **53033.3** | **5008.3** | **20300.0** | **1044.0** |
| **27.1** | **15100.0** | **2426.9** | **14033.3** | **305.5** | **16566.7** | **1686.2** | **15533.3** | **1040.8** | **17666.7** | **250.2** | **30566.7** | **6961.6** | **27800.0** | **2773.1** | **15766.7** | **986.6** | **18800.0** | **360.6** | **11300.0** | **100.0** | **17566.7** | **152.8** | **60500.0** | **2662.7** | **34800.0** | **2954.7** | **14433.3** | **57.7** |
| **28.1** | **10966.7** | **1501.1** | **9516.7** | **332.9** | **12133.3** | **802.1** | **9193.3** | **534.1** | **12866.7** | **250.2** | **21566.7** | **6269.2** | **18033.3** | **2375.6** | **11266.7** | **115.5** | **13566.7** | **115.5** | **7060.0** | **81.9** | **12600.0** | **264.6** | **38533.3** | **1803.7** | **21866.7** | **1514.4** | **10133.3** | **115.5** |
| **29.1** | **7240.0** | **983.2** | **6333.3** | **317.2** | **8273.3** | **596.5** | **5503.3** | **285.7** | **8836.7** | **5.1** | **15600.0** | **5640.0** | **11293.3** | **1746.5** | **7723.3** | **255.8** | **9761.7** | **163.6** | **4186.7** | **127.4** | **8593.3** | **297.0** | **22333.3** | **1305.1** | **14500.0** | **700.0** | **7080.0** | **60.8** |
| **30.1** | **4583.3** | **544.2** | **4200.0** | **230.7** | **5500.0** | **308.1** | **3486.7** | **150.4** | **6036.7** | **175.0** | **12026.7** | **5004.7** | **7480.0** | **1242.9** | **5026.7** | **250.1** | **7326.7** | **245.8** | **2470.0** | **95.4** | **5893.3** | **297.0** | **13800.0** | **1126.9** | **10800.0** | **173.2** | **5056.7** | **49.3** |
| **31.1** | **2896.7** | **315.6** | **2760.0** | **103.9** | **3653.3** | **155.7** | **2336.7** | **72.3** | **4096.7** | **125.0** | **9826.7** | **4310.8** | **5310.0** | **994.1** | **3213.3** | **168.6** | **5696.7** | **240.3** | **1446.7** | **40.4** | **3726.7** | **193.0** | **10243.3** | **1002.3** | **8326.7** | **186.1** | **3490.0** | **62.4** |
| **32.1** | **1800.0** | **202.2** | **1810.0** | **52.9** | **2406.7** | **106.9** | **1606.7** | **37.9** | **2746.7** | **55.0** | **8086.7** | **3307.3** | **3803.3** | **807.0** | **2065.0** | **109.0** | **4443.3** | **220.1** | **898.3** | **17.0** | **2376.7** | **130.5** | **8646.7** | **994.7** | **6440.0** | **399.6** | **2463.3** | **55.1** |
| **33.1** | **1206.7** | **122.2** | **1210.0** | **20.0** | **1643.3** | **97.1** | **1160.0** | **26.5** | **1850.0** | **10.0** | **6573.3** | **2451.4** | **2846.7** | **572.7** | **1311.3** | **70.9** | **3610.0** | **195.2** | **601.7** | **5.5** | **1503.3** | **83.3** | **7226.7** | **862.2** | **5086.7** | **480.5** | **1743.3** | **46.2** |
| **34.1** | **798.3** | **83.5** | **813.3** | **11.2** | **1136.7** | **80.2** | **870.3** | **22.5** | **1276.7** | **5.1** | **5000.0** | **793.8** | **2216.7** | **433.2** | **852.3** | **47.4** | **3023.3** | **182.3** | **437.3** | **6.4** | **951.7** | **50.5** | **6193.3** | **738.9** | **4246.7** | **539.0** | **1246.7** | **32.1** |
| **35.1** | **553.3** | **72.5** | **559.0** | **15.7** | **818.0** | **61.0** | **676.3** | **19.7** | **904.7** | **7.5** | **4283.3** | **90.2** | **1823.3** | **345.9** | **580.7** | **42.4** | **2616.7** | **181.5** | **335.7** | **11.5** | **604.3** | **24.4** | **5346.7** | **610.4** | **3723.3** | **574.0** | **875.0** | **24.3** |
| **36.1** | **397.3** | **65.9** | **394.0** | **26.5** | **617.0** | **40.1** | **552.0** | **19.2** | **675.7** | **1.5** | **4130.0** | **65.6** | **1570.0** | **278.4** | **423.3** | **44.7** | **2340.0** | **190.8** | **280.7** | **13.0** | **414.0** | **12.5** | **4783.3** | **520.8** | **3446.7** | **538.9** | **641.0** | **49.1** |
| **37.1** | **281.3** | **62.9** | **281.3** | **36.1** | **476.7** | **25.8** | **459.7** | **15.8** | **521.0** | **3.0** | **4133.3** | **51.3** | **1370.0** | **245.8** | **315.0** | **46.2** | **2003.3** | **176.2** | **226.7** | **17.5** | **302.7** | **6.8** | **4463.3** | **422.5** | **3363.3** | **518.1** | **491.3** | **79.4** |
| **38.1** | **226.3** | **42.8** | **215.3** | **37.7** | **413.0** | **14.8** | **385.3** | **11.2** | **447.0** | **2.0** | **4110.0** | **45.8** | **1400.0** | **221.1** | **240.7** | **42.4** | **1840.0** | **151.3** | **179.7** | **17.2** | **242.0** | **9.8** | **4416.7** | **297.7** | **3360.0** | **508.6** | **386.7** | **90.9** |
| **39.1** | **170.3** | **25.3** | **158.0** | **33.2** | **358.3** | **12.7** | **308.7** | **11.0** | **392.0** | **4.0** | **4143.3** | **40.4** | **1376.7** | **209.8** | **161.3** | **36.0** | **1746.7** | **181.5** | **130.0** | **19.3** | **199.0** | **15.1** | **4520.0** | **213.8** | **3360.0** | **488.7** | **308.7** | **107.2** |
| **40.1** | **133.7** | **16.8** | **120.3** | **26.4** | **314.3** | **15.3** | **228.3** | **18.9** | **346.7** | **8.5** | **4483.3** | **30.6** | **1426.7** | **221.9** | **97.5** | **20.9** | **1746.7** | **205.5** | **79.6** | **14.4** | **164.7** | **22.9** | **4633.3** | **170.4** | **3370.0** | **495.7** | **235.0** | **106.2** |
| **41.1** | **102.1** | **11.4** | **83.4** | **13.0** | **259.0** | **24.5** | **144.0** | **25.5** | **293.7** | **11.5** | **4866.7** | **55.1** | **1443.3** | **244.2** | **57.9** | **8.7** | **1823.3** | **220.3** | **50.8** | **5.0** | **127.5** | **26.8** | **4750.0** | **125.3** | **3436.7** | **491.2** | **175.6** | **88.2** |
| **42.1** | **68.6** | **7.2** | **56.0** | **5.8** | **179.7** | **19.6** | **55.7** | **8.8** | **216.0** | **6.0** | **4706.7** | **97.1** | **1157.3** | **223.9** | **37.6** | **6.0** | **1616.7** | **230.3** | **25.6** | **0.8** | **91.5** | **21.9** | **4973.3** | **105.0** | **3690.0** | **520.5** | **110.7** | **44.2** |
| **43.1** | **58.3** | **4.5** | **43.3** | **3.6** | **148.0** | **11.1** | **32.9** | **1.2** | **178.7** | **3.5** | **5280.0** | **50.0** | **1390.0** | **249.8** | **26.9** | **3.8** | **1903.3** | **318.8** | **10.2** | **0.5** | **61.2** | **7.9** | **5130.0** | **79.4** | **3996.7** | **499.4** | **66.7** | **15.7** |
| **44.1** | **44.7** | **4.0** | **33.7** | **2.4** | **120.0** | **8.2** | **21.7** | **0.4** | **147.7** | **1.5** | **5466.7** | **40.4** | **1416.7** | **251.1** | **19.3** | **2.2** | **2060.0** | **292.1** | **5.5** | **0.4** | **42.6** | **4.5** | **5233.3** | **66.6** | **4406.7** | **551.9** | **49.6** | **9.0** |
| **45.1** | **34.4** | **4.6** | **25.2** | **1.2** | **101.0** | **7.0** | **16.9** | **0.4** | **129.0** | **4.0** | **5666.7** | **40.4** | **1446.7** | **260.8** | **14.6** | **1.6** | **2210.0** | **270.6** | **4.4** | **0.2** | **28.3** | **2.8** | **4926.7** | **100.2** | **4690.0** | **531.1** | **35.7** | **4.7** |
| **46.1** | **27.3** | **3.7** | **18.3** | **0.3** | **83.3** | **4.7** | **14.9** | **0.3** | **110.7** | **4.5** | **5893.3** | **55.1** | **1513.3** | **237.1** | **11.3** | **1.2** | **2303.3** | **296.7** | **3.8** | **0.0** | **20.5** | **2.4** | **5206.7** | **98.7** | **4973.3** | **565.9** | **27.3** | **2.5** |
| **47.1** | **19.4** | **2.7** | **13.0** | **0.2** | **58.2** | **4.3** | **13.2** | **0.3** | **83.4** | **2.3** | **5366.7** | **317.9** | **1228.3** | **228.9** | **8.9** | **0.8** | **2226.7** | **287.5** | **3.4** | **0.0** | **17.0** | **3.1** | **5366.7** | **185.0** | **5386.7** | **597.7** | **22.8** | **0.9** |
| **48.1** | **19.2** | **2.6** | **10.3** | **0.2** | **53.7** | **5.6** | **12.1** | **0.3** | **82.4** | **0.4** | **5950.0** | **36.1** | **1526.7** | **169.2** | **7.6** | **0.5** | **2533.3** | **335.0** | **3.1** | **0.1** | **14.5** | **3.1** | **5333.3** | **250.1** | **5710.0** | **567.9** | **19.6** | **0.2** |
| **49.1** | **17.3** | **2.5** | **8.2** | **0.2** | **46.1** | **4.6** | **10.9** | **0.3** | **73.7** | **3.0** | **6130.0** | **65.6** | **1490.0** | **226.5** | **6.8** | **0.5** | **2603.3** | **344.9** | **2.7** | **0.0** | **13.9** | **3.9** | **5353.3** | **339.5** | **6060.0** | **589.5** | **17.5** | **0.1** |
| **50.1** | **16.2** | **2.5** | **6.7** | **0.1** | **40.8** | **4.8** | **9.8** | **0.3** | **69.6** | **4.4** | **6233.3** | **70.2** | **1653.3** | **221.2** | **6.0** | **0.5** | **2796.7** | **339.5** | **2.5** | **0.0** | **11.7** | **3.8** | **4846.7** | **359.5** | **5836.7** | **490.0** | **14.3** | **0.5** |
