# Supplementary material for: Refining the rheological characteristics of high drug loading ointment via SDS and machine learning
Source: PLoS One. 2024 May 9;19(5):e0303199. doi: 10.1371/journal.pone.0303199 (PMC11081290; doi:10.1371/journal.pone.0303199)
Supplement: S3 Table — (DOCX) [file pone.0303199.s006.docx]

| **Shear Rate**  **[1/s]** | **DoE 1** | | **DoE 2** | | **DoE 3** | | **DoE 4** | | **DoE 5** | | **DoE 6** | | **DoE 7** | | **DoE 8** | | **DoE 9** | | **DoE 10** | | **DoE 11** | | **DoE 12** | | **DoE 13** | | | **DoE 14** | |
| --- | --- | --- | --- | --- | --- | --- | --- | --- | --- | --- | --- | --- | --- | --- | --- | --- | --- | --- | --- | --- | --- | --- | --- | --- | --- | --- | --- | --- | --- |
|  | **Mean** | **SD** | **Mean** | **SD** | **Mean** | **SD** | **Mean** | **SD** | **Mean** | **SD** | **Mean** | **SD** | **Mean** | **SD** | **Mean** | **SD** | **Mean** | **SD** | **Mean** | **SD** | **Mean** | **SD** | **Mean** | **SD** | | **Mean** | **SD** | **Mean** | **SD** |
| **0.01** | **9963.3** | **671.6** | **11900.0** | **1276.7** | **15966.7** | **907.4** | **17233.3** | **832.7** | **19666.7** | **2010.8** | **32466.7** | **3764.7** | **21733.3** | **929.2** | **12633.3** | **1006.6** | **17866.7** | **1209.7** | **7166.7** | **378.7** | **16166.7** | **907.4** | **21833.3** | **550.8** | | **34750.0** | **1650.0** | **13966.7** | **1101.5** |
| **0.0147** | **6906.7** | **739.0** | **8653.3** | **1167.3** | **14500.0** | **100.0** | **13133.3** | **723.4** | **18533.3** | **1464.0** | **30933.3** | **1800.9** | **21700.0** | **1044.0** | **11833.3** | **1115.0** | **15000.0** | **1081.7** | **5170.0** | **78.1** | **13000.0** | **721.1** | **24466.7** | **513.2** | | **31550.0** | **150.0** | **11466.7** | **1193.0** |
| **0.0215** | **4523.3** | **396.3** | **5363.3** | **514.8** | **10050.0** | **412.4** | **9166.7** | **817.3** | **14366.7** | **1594.8** | **26000.0** | **916.5** | **18266.7** | **1159.0** | **8773.3** | **241.7** | **10593.3** | **805.1** | **3676.7** | **66.6** | **8966.7** | **500.1** | **22733.3** | **208.2** | | **24700.0** | **600.0** | **8463.3** | **972.2** |
| **0.0316** | **3016.7** | **205.5** | **3466.7** | **173.9** | **6576.7** | **394.0** | **6370.0** | **800.2** | **10123.3** | **1372.1** | **20466.7** | **416.3** | **13733.3** | **1209.7** | **5920.0** | **538.4** | **7080.0** | **571.9** | **2656.7** | **23.1** | **5836.7** | **357.3** | **18000.0** | **624.5** | | **17800.0** | **700.0** | **6000.0** | **701.9** |
| **0.0464** | **2063.3** | **158.9** | **2393.3** | **100.7** | **4363.3** | **310.2** | **4453.3** | **657.3** | **6963.3** | **1018.6** | **15400.0** | **360.6** | **9563.3** | **1119.2** | **3926.7** | **698.9** | **4656.7** | **400.8** | **1936.7** | **64.3** | **3780.0** | **181.9** | **12600.0** | **781.0** | | **12050.0** | **550.0** | **4170.0** | **475.1** |
| **0.0681** | **1443.3** | **153.1** | **1726.7** | **81.4** | **2943.3** | **257.0** | **3136.7** | **477.5** | **4823.3** | **784.4** | **11133.3** | **208.2** | **6346.7** | **925.0** | **2613.3** | **557.2** | **3060.0** | **272.2** | **1413.3** | **92.9** | **2533.3** | **66.6** | **8493.3** | **647.3** | | **7900.0** | **350.0** | **2853.3** | **312.1** |
| **0.1** | **1037.0** | **146.9** | **1276.7** | **55.1** | **2016.7** | **195.5** | **2250.0** | **321.9** | **3346.7** | **587.9** | **7880.0** | **125.3** | **4146.7** | **691.7** | **1763.3** | **366.7** | **2030.0** | **185.2** | **1046.7** | **98.2** | **1766.7** | **11.5** | **5586.7** | **498.1** | | **5100.0** | **190.0** | **1943.3** | **213.9** |
| **0.147** | **748.0** | **119.9** | **945.3** | **29.0** | **1390.0** | **151.3** | **1650.0** | **210.7** | **2293.3** | **346.7** | **5510.0** | **86.6** | **2716.7** | **517.3** | **1197.7** | **212.1** | **1390.0** | **127.7** | **772.0** | **95.3** | **1260.0** | **10.0** | **3703.3** | **336.2** | | **3285.0** | **85.0** | **1320.0** | **141.8** |
| **0.215** | **537.7** | **87.5** | **697.3** | **12.9** | **980.0** | **105.8** | **1240.0** | **125.3** | **1616.7** | **210.8** | **3806.7** | **47.3** | **1806.7** | **392.7** | **817.0** | **116.5** | **987.3** | **89.7** | **571.0** | **95.3** | **913.7** | **19.5** | **2480.0** | **249.8** | | **2130.0** | **30.0** | **892.0** | **86.5** |
| **0.316** | **385.7** | **61.3** | **519.0** | **5.6** | **716.7** | **71.5** | **949.7** | **69.0** | **1156.7** | **107.9** | **2603.3** | **41.6** | **1213.3** | **300.4** | **570.0** | **56.2** | **733.3** | **63.0** | **423.7** | **94.7** | **667.7** | **19.5** | **1710.0** | **177.8** | | **1390.0** | **0.0** | **600.7** | **54.5** |
| **0.464** | **280.0** | **47.3** | **395.3** | **1.5** | **543.7** | **48.0** | **737.7** | **27.5** | **839.3** | **66.5** | **1773.3** | **32.1** | **832.0** | **232.9** | **406.7** | **20.2** | **567.7** | **40.4** | **317.3** | **95.9** | **486.7** | **18.6** | **1200.0** | **137.5** | | **917.5** | **14.5** | **403.7** | **33.0** |
| **0.681** | **211.7** | **41.0** | **305.3** | **5.0** | **422.0** | **30.4** | **575.7** | **4.6** | **605.7** | **43.4** | **1203.3** | **23.1** | **576.7** | **178.0** | **295.7** | **6.7** | **454.7** | **17.2** | **242.7** | **100.8** | **346.7** | **17.8** | **872.3** | **101.5** | | **613.5** | **18.5** | **269.0** | **21.0** |
| **1** | **167.7** | **38.7** | **229.0** | **11.5** | **324.0** | **17.4** | **429.0** | **5.0** | **438.0** | **38.4** | **808.0** | **18.5** | **406.7** | **135.7** | **215.7** | **9.5** | **366.7** | **15.0** | **189.0** | **101.3** | **244.7** | **12.2** | **662.7** | **75.6** | | **416.5** | **18.5** | **180.0** | **14.7** |
| **1.47** | **134.3** | **32.5** | **170.3** | **9.9** | **244.3** | **5.5** | **305.7** | **4.9** | **314.7** | **26.0** | **539.3** | **11.5** | **290.7** | **102.9** | **155.3** | **6.0** | **279.7** | **6.8** | **134.7** | **75.6** | **179.7** | **6.0** | **539.7** | **58.5** | | **288.5** | **13.5** | **121.3** | **10.7** |
| **2.15** | **107.7** | **23.7** | **131.3** | **7.0** | **181.0** | **5.3** | **210.0** | **4.4** | **218.3** | **11.0** | **359.3** | **11.0** | **212.0** | **76.7** | **113.3** | **5.5** | **202.3** | **4.5** | **94.6** | **52.3** | **137.0** | **2.0** | **459.3** | **21.2** | | **205.0** | **8.0** | **83.1** | **7.0** |
| **3.16** | **87.6** | **13.0** | **104.3** | **2.5** | **127.7** | **6.4** | **144.0** | **3.0** | **145.3** | **5.1** | **241.0** | **10.5** | **157.0** | **56.0** | **82.2** | **3.9** | **144.3** | **2.5** | **66.3** | **35.3** | **105.7** | **0.6** | **317.0** | **42.8** | | **148.0** | **5.0** | **57.5** | **4.3** |
| **4.64** | **67.7** | **2.7** | **72.9** | **0.4** | **86.3** | **4.5** | **100.3** | **1.6** | **96.9** | **3.8** | **162.7** | **3.5** | **117.2** | **37.8** | **58.6** | **2.1** | **103.0** | **2.0** | **47.3** | **23.7** | **80.3** | **1.1** | **189.3** | **14.4** | | **109.0** | **2.0** | **40.6** | **2.5** |
| **6.81** | **47.8** | **4.3** | **47.4** | **0.8** | **57.9** | **2.4** | **70.5** | **0.9** | **66.5** | **0.4** | **107.0** | **1.0** | **87.9** | **21.8** | **42.1** | **0.8** | **74.1** | **1.0** | **34.1** | **15.7** | **59.6** | **0.6** | **119.0** | **1.7** | | **82.8** | **1.1** | **29.2** | **1.4** |
| **10** | **33.4** | **3.4** | **31.5** | **1.4** | **38.7** | **1.9** | **50.1** | **0.6** | **43.8** | **0.8** | **74.3** | **1.4** | **63.6** | **12.5** | **30.4** | **0.4** | **53.5** | **0.9** | **25.2** | **10.0** | **42.7** | **0.6** | **83.7** | **1.8** | | **65.0** | **0.4** | **21.7** | **0.6** |
| **14.7** | **23.4** | **2.7** | **20.8** | **1.0** | **26.6** | **1.9** | **36.1** | **0.9** | **29.7** | **1.2** | **50.5** | **1.9** | **45.8** | **5.6** | **22.0** | **0.6** | **38.8** | **0.8** | **19.1** | **6.3** | **29.7** | **0.8** | **56.9** | **0.6** | | **53.3** | **0.3** | **16.4** | **0.3** |
| **21.5** | **16.6** | **1.9** | **14.2** | **1.0** | **19.0** | **1.4** | **26.4** | **0.2** | **20.8** | **1.2** | **36.3** | **1.2** | **33.4** | **1.8** | **15.9** | **0.6** | **28.7** | **0.4** | **14.9** | **3.7** | **20.7** | **1.2** | **40.4** | **0.7** | | **43.6** | **0.2** | **12.8** | **0.1** |
| **31.6** | **12.0** | **1.3** | **9.9** | **0.8** | **14.0** | **1.4** | **19.3** | **0.1** | **14.4** | **0.9** | **26.1** | **1.2** | **23.6** | **1.5** | **11.2** | **0.5** | **21.2** | **0.3** | **11.4** | **2.2** | **14.7** | **0.7** | **29.1** | **0.8** | | **33.5** | **0.4** | **10.4** | **0.1** |
| **46.4** | **8.8** | **0.9** | **7.1** | **0.6** | **10.3** | **1.7** | **14.0** | **0.0** | **10.5** | **0.4** | **19.0** | **1.2** | **16.7** | **1.4** | **8.1** | **0.3** | **15.7** | **0.3** | **8.5** | **1.5** | **10.6** | **0.5** | **21.1** | **0.9** | | **23.3** | **0.6** | **8.6** | **0.0** |
| **68.1** | **6.5** | **0.7** | **5.0** | **0.5** | **7.6** | **1.8** | **10.2** | **0.0** | **7.6** | **0.6** | **14.0** | **1.1** | **12.1** | **1.2** | **5.9** | **0.2** | **11.9** | **0.3** | **6.3** | **1.1** | **7.5** | **0.4** | **15.5** | **0.8** | | **16.1** | **0.1** | **7.1** | **0.1** |
| **100** | **4.9** | **0.5** | **3.6** | **0.4** | **5.8** | **1.4** | **7.5** | **0.0** | **5.6** | **0.5** | **10.6** | **0.8** | **9.0** | **0.9** | **4.3** | **0.2** | **9.2** | **0.2** | **4.7** | **0.9** | **5.4** | **0.3** | **11.9** | **0.4** | | **10.5** | **0.3** | **4.6** | **0.5** |

**S3 Table. Viscosity (Pa.s) and shear rate (s-1) data of DoE formula (*n*=3)**
