# Supplementary material for: Refining the rheological characteristics of high drug loading ointment via SDS and machine learning
Source: PLoS One. 2024 May 9;19(5):e0303199. doi: 10.1371/journal.pone.0303199 (PMC11081290; doi:10.1371/journal.pone.0303199)
Supplement: S4 Table — (DOCX) [file pone.0303199.s007.docx]

**S4 Table. Summary of 128 simulation experiments**

| **模拟次数** | **X1** | **X2** | **X3** | **X4** | **X5** | **总缺陷率** | **Log10 缺陷率** |
| --- | --- | --- | --- | --- | --- | --- | --- |
| **1** | **32.99** | **18.86** | **1479.48** | **76.41** | **6.99** | **0.82** | **-0.08** |
| **2** | **23.54** | **9.25** | **1231.45** | **79.19** | **6.12** | **0.21** | **-0.69** |
| **3** | **28.27** | **8.46** | **818.06** | **76.29** | **7.22** | **0.38** | **-0.42** |
| **4** | **20.35** | **5.79** | **1284.60** | **73.70** | **5.55** | **0.80** | **-0.10** |
| **5** | **20.12** | **7.36** | **1349.56** | **73.64** | **6.97** | **0.66** | **-0.18** |
| **6** | **34.76** | **12.56** | **900.74** | **75.11** | **7.62** | **0.90** | **-0.04** |
| **7** | **33.82** | **13.98** | **1526.72** | **74.94** | **5.96** | **0.84** | **-0.08** |
| **8** | **29.80** | **14.92** | **1148.77** | **80.49** | **7.20** | **0.45** | **-0.35** |
| **9** | **25.20** | **14.45** | **1467.67** | **73.58** | **6.08** | **0.54** | **-0.27** |
| **10** | **34.53** | **7.68** | **1538.54** | **80.25** | **6.57** | **0.73** | **-0.13** |
| **11** | **30.39** | **6.73** | **1266.88** | **77.65** | **7.48** | **0.62** | **-0.21** |
| **12** | **34.29** | **23.11** | **877.12** | **80.61** | **5.14** | **0.94** | **-0.03** |
| **13** | **30.75** | **15.87** | **1184.20** | **80.31** | **5.35** | **0.59** | **-0.23** |
| **14** | **20.47** | **11.61** | **847.59** | **77.83** | **5.94** | **0.43** | **-0.36** |
| **15** | **34.41** | **22.95** | **1107.43** | **78.42** | **6.63** | **0.87** | **-0.06** |
| **16** | **24.84** | **10.98** | **1119.24** | **75.64** | **5.86** | **0.35** | **-0.46** |
| **17** | **22.01** | **12.72** | **871.21** | **73.93** | **6.14** | **0.52** | **-0.29** |
| **18** | **32.87** | **20.59** | **1201.92** | **79.48** | **6.34** | **0.75** | **-0.13** |
| **19** | **30.16** | **23.90** | **1455.86** | **74.46** | **6.95** | **0.78** | **-0.11** |
| **20** | **29.45** | **14.13** | **1131.06** | **79.60** | **5.84** | **0.36** | **-0.44** |
| **21** | **24.37** | **16.81** | **1249.17** | **77.12** | **7.34** | **0.40** | **-0.40** |
| **22** | **25.43** | **16.18** | **1136.96** | **76.94** | **7.01** | **0.35** | **-0.45** |
| **23** | **22.83** | **13.82** | **1255.07** | **79.31** | **6.77** | **0.26** | **-0.59** |
| **24** | **21.18** | **21.22** | **1083.81** | **73.22** | **5.71** | **0.63** | **-0.20** |
| **25** | **24.96** | **17.28** | **1473.57** | **75.05** | **5.33** | **0.42** | **-0.37** |
| **26** | **35.00** | **11.93** | **1012.94** | **74.35** | **6.79** | **0.89** | **-0.05** |
| **27** | **31.46** | **20.12** | **1160.58** | **73.28** | **5.45** | **0.86** | **-0.07** |
| **28** | **23.07** | **15.55** | **959.80** | **78.13** | **6.18** | **0.32** | **-0.50** |
| **29** | **21.89** | **15.71** | **1219.64** | **77.00** | **5.25** | **0.30** | **-0.52** |
| **30** | **31.34** | **9.72** | **1550.35** | **74.17** | **6.91** | **0.81** | **-0.09** |
| **31** | **31.69** | **5.94** | **1444.05** | **78.83** | **7.18** | **0.64** | **-0.19** |
| **32** | **31.93** | **7.05** | **1272.79** | **74.64** | **6.83** | **0.80** | **-0.09** |
| **33** | **32.05** | **17.76** | **1408.61** | **77.89** | **7.16** | **0.72** | **-0.14** |
| **34** | **34.88** | **15.08** | **1337.75** | **73.87** | **5.17** | **0.92** | **-0.04** |
| **35** | **23.43** | **22.17** | **1432.24** | **79.42** | **5.47** | **0.65** | **-0.19** |
| **36** | **23.31** | **16.50** | **1007.04** | **80.01** | **7.26** | **0.48** | **-0.32** |
| **37** | **21.06** | **15.39** | **1509.01** | **78.01** | **6.45** | **0.31** | **-0.51** |
| **38** | **34.17** | **7.52** | **812.16** | **76.77** | **6.47** | **0.57** | **-0.24** |
| **39** | **22.48** | **23.27** | **1036.57** | **74.29** | **7.06** | **0.76** | **-0.12** |
| **40** | **20.24** | **15.24** | **1390.90** | **75.29** | **6.06** | **0.34** | **-0.47** |
| **41** | **24.72** | **8.31** | **1060.19** | **77.30** | **7.30** | **0.24** | **-0.61** |
| **42** | **20.94** | **12.87** | **1296.41** | **78.89** | **5.98** | **0.31** | **-0.51** |
| **43** | **33.11** | **14.61** | **1066.09** | **73.99** | **7.05** | **0.87** | **-0.06** |
| **44** | **33.58** | **19.17** | **1355.46** | **77.42** | **5.92** | **0.79** | **-0.10** |
| **45** | **27.80** | **10.51** | **947.98** | **74.52** | **5.63** | **0.51** | **-0.30** |
| **46** | **28.39** | **6.89** | **942.08** | **76.53** | **6.43** | **0.30** | **-0.52** |
| **47** | **32.76** | **6.57** | **1278.69** | **78.48** | **6.04** | **0.43** | **-0.37** |
| **48** | **29.92** | **24.84** | **1402.71** | **75.82** | **6.73** | **0.75** | **-0.12** |
| **49** | **23.19** | **20.91** | **1308.22** | **78.24** | **7.24** | **0.57** | **-0.25** |
| **50** | **24.25** | **19.02** | **1207.83** | **80.72** | **5.53** | **0.62** | **-0.21** |
| **51** | **25.79** | **8.78** | **965.70** | **80.07** | **5.88** | **0.30** | **-0.52** |
| **52** | **27.32** | **9.09** | **1072.00** | **74.76** | **7.38** | **0.62** | **-0.21** |
| **53** | **33.35** | **21.85** | **865.31** | **78.77** | **7.28** | **0.79** | **-0.10** |
| **54** | **26.61** | **20.43** | **1125.15** | **78.18** | **5.49** | **0.46** | **-0.33** |
| **55** | **27.20** | **5.00** | **1290.50** | **76.88** | **5.37** | **0.39** | **-0.41** |
| **56** | **30.63** | **18.39** | **1485.39** | **77.71** | **6.36** | **0.62** | **-0.21** |
| **57** | **27.56** | **13.50** | **971.61** | **74.05** | **6.38** | **0.57** | **-0.24** |
| **58** | **21.77** | **10.35** | **1095.62** | **75.47** | **6.89** | **0.40** | **-0.39** |
| **59** | **23.78** | **18.54** | **930.27** | **77.95** | **7.52** | **0.57** | **-0.25** |
| **60** | **25.67** | **5.16** | **1190.11** | **75.17** | **6.22** | **0.53** | **-0.27** |
| **61** | **33.94** | **5.47** | **1343.65** | **79.66** | **5.19** | **0.59** | **-0.23** |
| **62** | **32.64** | **13.19** | **995.23** | **76.65** | **5.61** | **0.52** | **-0.28** |
| **63** | **29.57** | **10.83** | **1018.85** | **76.18** | **6.75** | **0.44** | **-0.35** |
| **64** | **23.66** | **18.07** | **829.87** | **74.23** | **5.77** | **0.46** | **-0.34** |
| **65** | **32.28** | **8.94** | **1101.53** | **77.77** | **6.42** | **0.46** | **-0.34** |
| **66** | **33.23** | **16.34** | **1532.63** | **78.60** | **5.65** | **0.76** | **-0.12** |
| **67** | **34.06** | **9.57** | **936.17** | **79.37** | **6.49** | **0.56** | **-0.25** |
| **68** | **26.14** | **19.33** | **1001.13** | **76.35** | **5.43** | **0.40** | **-0.40** |
| **69** | **31.22** | **19.96** | **989.32** | **77.24** | **7.40** | **0.68** | **-0.17** |
| **70** | **22.72** | **24.53** | **1361.37** | **80.55** | **7.03** | **0.78** | **-0.11** |
| **71** | **33.70** | **21.54** | **1414.52** | **75.70** | **6.20** | **0.88** | **-0.06** |
| **72** | **28.15** | **20.28** | **1396.80** | **78.54** | **7.60** | **0.56** | **-0.25** |
| **73** | **22.13** | **10.20** | **1384.99** | **78.07** | **7.58** | **0.23** | **-0.64** |
| **74** | **29.21** | **9.88** | **1514.91** | **80.37** | **7.08** | **0.47** | **-0.33** |
| **75** | **25.91** | **25.00** | **853.50** | **80.43** | **6.85** | **0.79** | **-0.10** |
| **76** | **31.57** | **22.32** | **1373.18** | **73.81** | **5.31** | **0.88** | **-0.06** |
| **77** | **29.09** | **24.69** | **841.69** | **77.59** | **5.80** | **0.74** | **-0.13** |
| **78** | **30.87** | **22.64** | **983.42** | **75.35** | **7.54** | **0.77** | **-0.11** |
| **79** | **25.31** | **16.02** | **924.36** | **79.72** | **5.51** | **0.48** | **-0.32** |
| **80** | **27.68** | **11.46** | **1154.68** | **75.59** | **5.82** | **0.39** | **-0.41** |
| **81** | **24.49** | **13.66** | **1243.26** | **79.13** | **6.61** | **0.22** | **-0.65** |
| **82** | **20.59** | **12.40** | **1237.35** | **79.90** | **5.67** | **0.46** | **-0.33** |
| **83** | **30.98** | **10.04** | **977.51** | **78.95** | **5.23** | **0.37** | **-0.43** |
| **84** | **22.36** | **6.42** | **1113.34** | **76.06** | **6.59** | **0.37** | **-0.43** |
| **85** | **21.42** | **17.60** | **1544.44** | **77.53** | **7.14** | **0.40** | **-0.40** |
| **86** | **31.10** | **6.10** | **894.83** | **77.36** | **7.50** | **0.59** | **-0.23** |
| **87** | **26.26** | **19.80** | **1042.47** | **75.00** | **5.57** | **0.49** | **-0.31** |
| **88** | **20.71** | **17.44** | **800.35** | **75.76** | **7.56** | **0.62** | **-0.21** |
| **89** | **33.46** | **22.80** | **835.78** | **79.96** | **6.10** | **0.86** | **-0.06** |
| **90** | **20.83** | **23.74** | **1048.38** | **73.40** | **7.36** | **0.86** | **-0.06** |
| **91** | **21.30** | **7.20** | **1030.66** | **76.71** | **5.59** | **0.50** | **-0.30** |
| **92** | **26.02** | **7.83** | **1426.33** | **74.82** | **6.53** | **0.48** | **-0.32** |
| **93** | **29.69** | **8.15** | **888.93** | **78.72** | **5.75** | **0.27** | **-0.56** |
| **94** | **25.08** | **16.97** | **1461.76** | **79.25** | **5.16** | **0.54** | **-0.27** |
| **95** | **24.61** | **23.58** | **1302.31** | **75.23** | **6.32** | **0.58** | **-0.24** |
| **96** | **29.33** | **21.06** | **1196.02** | **78.30** | **5.39** | **0.60** | **-0.22** |
| **97** | **28.86** | **13.35** | **1420.43** | **79.01** | **6.16** | **0.36** | **-0.44** |
| **98** | **30.28** | **12.09** | **1166.49** | **73.76** | **6.28** | **0.75** | **-0.12** |
| **99** | **32.52** | **5.31** | **859.40** | **74.58** | **6.00** | **0.62** | **-0.21** |
| **100** | **23.90** | **17.13** | **1225.54** | **74.11** | **6.24** | **0.53** | **-0.28** |
| **101** | **32.40** | **12.24** | **1054.28** | **76.47** | **7.44** | **0.75** | **-0.13** |
| **102** | **22.60** | **18.70** | **1449.95** | **79.07** | **7.46** | **0.52** | **-0.28** |
| **103** | **27.91** | **13.03** | **1367.28** | **73.46** | **7.10** | **0.74** | **-0.13** |
| **104** | **26.73** | **18.23** | **1320.03** | **76.59** | **6.65** | **0.40** | **-0.39** |
| **105** | **27.44** | **17.91** | **1178.30** | **75.94** | **6.40** | **0.44** | **-0.36** |
| **106** | **22.24** | **16.65** | **823.97** | **80.13** | **6.67** | **0.57** | **-0.25** |
| **107** | **28.03** | **20.75** | **806.25** | **78.66** | **5.21** | **0.66** | **-0.18** |
| **108** | **20.00** | **14.76** | **1497.20** | **79.54** | **5.27** | **0.61** | **-0.21** |
| **109** | **30.04** | **22.48** | **1260.98** | **77.06** | **7.32** | **0.67** | **-0.18** |
| **110** | **28.74** | **24.06** | **1331.84** | **73.52** | **5.79** | **0.80** | **-0.09** |
| **111** | **32.17** | **19.65** | **1503.10** | **74.40** | **5.41** | **0.85** | **-0.07** |
| **112** | **24.13** | **9.41** | **1520.82** | **78.36** | **6.26** | **0.22** | **-0.65** |
| **113** | **30.51** | **23.43** | **1077.91** | **80.66** | **5.29** | **0.85** | **-0.07** |
| **114** | **26.97** | **8.62** | **912.55** | **76.00** | **6.87** | **0.34** | **-0.47** |
| **115** | **26.85** | **11.30** | **1379.09** | **79.78** | **7.12** | **0.28** | **-0.55** |
| **116** | **24.02** | **24.21** | **1024.76** | **77.48** | **6.71** | **0.68** | **-0.17** |
| **117** | **28.98** | **14.29** | **1314.13** | **76.83** | **5.12** | **0.42** | **-0.38** |
| **118** | **28.62** | **24.37** | **953.89** | **77.18** | **6.69** | **0.69** | **-0.16** |
| **119** | **26.38** | **21.38** | **1325.94** | **75.41** | **6.81** | **0.55** | **-0.26** |
| **120** | **28.50** | **19.49** | **906.65** | **73.34** | **5.90** | **0.69** | **-0.16** |
| **121** | **21.54** | **10.67** | **918.46** | **76.12** | **5.73** | **0.40** | **-0.39** |
| **122** | **31.81** | **6.26** | **1172.39** | **80.19** | **6.55** | **0.36** | **-0.45** |
| **123** | **26.50** | **7.99** | **1491.29** | **74.88** | **7.42** | **0.59** | **-0.23** |
| **124** | **21.65** | **11.77** | **883.02** | **79.84** | **6.93** | **0.36** | **-0.44** |
| **125** | **27.09** | **22.01** | **1142.87** | **74.70** | **5.69** | **0.60** | **-0.22** |
| **126** | **22.95** | **5.63** | **1213.73** | **76.24** | **6.02** | **0.41** | **-0.39** |
| **127** | **25.55** | **21.69** | **1438.14** | **75.53** | **6.30** | **0.50** | **-0.31** |
| **128** | **34.65** | **11.14** | **1089.72** | **75.88** | **6.51** | **0.78** | **-0.11** |
